# Identification and Transcript Analysis of the TCP Transcription Factors in the Diploid Woodland Strawberry *Fragaria vesca*

**DOI:** 10.3389/fpls.2016.01937

**Published:** 2016-12-22

**Authors:** Wei Wei, Yang Hu, Meng-Yuan Cui, Yong-Tao Han, Kuan Gao, Jia-Yue Feng

**Affiliations:** ^1^State Key Laboratory of Crop Stress Biology for Arid Areas, College of Horticulture, Northwest A&F UniversityShaanxi, China; ^2^Key Laboratory of Protected Horticulture Engineering in Northwest China, Ministry of AgricultureShaanxi, China

**Keywords:** Strawberry (*Fragaria vesca*), TCP transcription factors, plant growth and development, transcript accumulation profiles, subcellular localization, transient over-expression

## Abstract

Plant-specific TEOSINTE BRANCHED 1, CYCLOIDEA, and PROLIFERATING CELL FACTORS (TCP) transcription factors play versatile functions in multiple processes of plant growth and development. However, no systematic study has been performed in strawberry. In this study, 19 *FvTCP* genes were identified in the diploid woodland strawberry (*Fragaria vesca*) accession Heilongjiang-3. Phylogenetic analysis suggested that the *FvTCP* genes were classified into two main classes, with the second class further divided into two subclasses, which was supported by the exon-intron organizations and the conserved motif structures. Promoter analysis revealed various *cis*-acting elements related to growth and development, hormone and/or stress responses. We analyzed *FvTCP* gene transcript accumulation patterns in different tissues and fruit developmental stages. Among them, 12 *FvTCP* genes exhibited distinct tissue-specific transcript accumulation patterns. Eleven *FvTCP* genes were down-regulated in different fruit developmental stages, while five *FvTCP* genes were up-regulated. Transcripts of *FvTCP* genes also varied with different subcultural propagation periods and were induced by hormone treatments and biotic and abiotic stresses. Subcellular localization analysis showed that six FvTCP-GFP fusion proteins showed distinct localizations in *Arabidopsis* mesophyll protoplasts. Notably, transient over-expression of *FvTCP9* in strawberry fruits dramatically affected the expression of a series of genes implicated in fruit development and ripening. Taken together, the present study may provide the basis for functional studies to reveal the role of this gene famil*y* in strawberry growth and development.

## Introduction

TEOSINTE BRANCHED 1, CYCLOIDEA, and PROLIFERATING CELL FACTORS (TCP) transcription factors constitute a small family of plant-specific transcription factors that play versatile functions in regulating diverse plant growth and development processes by controlling cell proliferation (Martin-Trillo and Cubas, 2010). TCP transcription factors were discovered in 1999 and named after the first three characterized family members: TEOSINTE BRANCHED 1 (TB1) in maize (*Zea mays*), CYCLOIDEA (CYC) in snapdragon (*Antirrhinum majus*), and PROLIFERATING CELL FACTORS 1 and 2 (PCF1 and PCF2) in rice (*Oryza sativa*; Cubas et al., 1999). This class of transcription factors is characterized by a highly conserved 59-residue-long non-canonical basic helix-loop-helix (bHLH) structure at the N-terminus called the TCP domain, which is involved in DNA binding, protein nuclear localization, and protein–protein interactions (Cubas et al., 1999; Kosugi and Ohashi, 2002). According to the homology of the TCP domains, the members of the TCP family can be divided into two classes: class I (also named PCF or TCP-P) and class II (also named TCP-C; Navaud et al., 2007). The most striking difference between these two classes is a four-amino-acid deletion in the basic region of the TCP domain of class I relative to class II proteins (Martin-Trillo and Cubas, 2010). The members of class II are quite heterogeneous and can be further subdivided into the CIN and CYC/TB1 subclades (Martin-Trillo and Cubas, 2010). Outside the TCP domain, several class II members are present in an 18-20-residue arginine-rich motif called the R domain with an unknown function, which is speculated to facilitate protein–protein interactions (Cubas et al., 1999).

TCP proteins play a versatile function in multiple biological processes during plant growth and development. It has been reported that many TCP transcription factors participate in the regulation of diverse physiological and biological processes, such as branching (Takeda et al., 2003; Aguilar-Martinez et al., 2007), leaf development (Kieffer et al., 2011), flower development (Kieffer et al., 2011), hormone pathways (Aguilar-Martinez et al., 2007), seed germination (Tatematsu et al., 2008), gametophyte development (Pagnussat et al., 2005), mitochondrial biogenesis (Hammani et al., 2011), and regulation of the circadian clock (Giraud et al., 2010) in various plants. The CIN-like clade genes are involved in lateral organ development, and the CYC/TB1 clade genes control axillary meristem development. In *Arabidopsis*, functional analysis of two homologs of *TB1*, *BRANCHED1* (*BRC1*, *AtTCP18*) and *BRANCHED2* (*BRC2*, *AtTCP12*), demonstrated that these genes were involved in suppressing axillary bud outgrowth (Aguilar-Martinez et al., 2007). Five CIN-like *TCP* genes (*TCP2*, *TCP3*, *TCP4*, *TCP10*, and *TCP24*) in *Arabidopsis* were all targeted by miR319 and have been implicated in regulating leaf morphogenesis (Nath et al., 2003; Schommer et al., 2008). By contrast, during plant development, class I *TCP* genes mainly promote cell growth and proliferation (Kosugi and Ohashi, 2002; Danisman et al., 2012). Recently, experimental evidence has shown that TCP proteins could be involved in fruit development and ripening (Parapunova et al., 2014).

Recently, a number of TCP proteins have been identified in various plants due to completion of their whole genome sequence, such as *Arabidopsis thaliana* (Riechmann et al., 2000), rice (*O. sativa*; Yao et al., 2007), tomato (*Solanum lycopersicum*; Parapunova et al., 2014), apple (*Malus domestica*; Xu et al., 2014), cotton (*Gossypium raimondii*; Ma et al., 2014), and watermelon (*Citrullus lanatus*; Shi et al., 2016). However, among the largest and most diverse gene families, the *TCP* gene family has not been systematically identified in the strawberry genome. To date, only the strawberry *FaTCP11* gene has been shown to play a role in ripening and in the regulation of flavan-3-ols synthesis (Pillet et al., 2015).

The cultivated strawberry (*Fragaria* × *ananassa* Duch.), which has great nutritive and commercial value, is one of the important horticultural crops grown worldwide for the production of fresh fruit and juice, among other products, and is also an excellent model plant for fleshy fruit development. *F.* × *ananassa* has a relatively complex octoploid genome, harboring 56 chromosomes (2*n* = 8*x* = 56) that were likely derived from four diploid ancestors (Kang and Liu, 2015). Thus, the sequenced diploid woodland strawberry *Fragaria vesca* accession Hawaii-4 with a small genome (240 Mb genome, 2*n* = 2*x* = 14) offers the possibility of a genome-wide analysis of *TCP* genes (Shulaev et al., 2011). ‘Heilongjiang-3’ strawberry, from the Heilongjiang province in China, was identified as the diploid woodland strawberry *F. vesca* (Lei et al., 1997).

In the present study, 19 *TCP* genes were identified in the diploid woodland strawberry (*F. vesca*) accession Heilongjiang-3, and a systematic bioinformatics analysis was performed, including determination of the linkage group location, phylogenetic relationships, gene structure, protein motifs and *cis*-acting elements. We further characterized the transcript accumulation patterns of *FvTCP* genes in diverse tissues, different stages of fruit development and ripening, different periods of strawberry subcultural propagation, as well in response to hormones and stress treatment. Additionally, we determined the subcellular localization of six FvTCP proteins in *Arabidopsis* mesophyll protoplasts and transiently over-expressed *FvTCP9* in strawberry fruits via agro-infiltration. This study provides details regarding the *TCP* gene family and facilitates the further functional characterization of *TCP* genes in strawberry.

## Materials and Methods

### Plant Materials, Growth Conditions, and Stress Treatments

The wild diploid strawberry *F. vesca* accession Heilongjiang-3 was obtained from the strawberry germplasm resource greenhouse of the College of Horticulture, Northwest A&F University, Shaanxi, Yangling, China (34° 20′ N 108° 24′ E). The potted strawberry plants were grown at 22°C with 75% relative humidity and no supplemental light. ‘Heilongjiang-3’ strawberry organs/tissues (roots, stems, runners, leaves, floral buds, flowers, mature flowers with partially withered petals, mature green receptacles, white receptacles with green achenes, half white and half red fruits, and fully ripened fruits) were collected for tissue-specific and different developmental stages of the fruits were collected for stage-specific transcript analysis of the *FvTCP* genes. The strawberry tissue culture plantlets were transferred to proliferation medium consisting of an MS basal medium supplemented with 30 g L^-1^ sucrose, 7 g L^-1^ agar, 0.2 mg L^-1^ 6-benzyladenine (6-BA) and 0.8 mg L^-1^ indole- 3-butyric acid (IBA) with monthly subculturing for induction and the following five different subcultural propagation periods (P1: original plantlet; P2: plantlet with 1–2 branch crowns, approximately 2 weeks after subculture; P3: plantlets with 3–4 branch crowns, approximately 3 weeks after subculture; P4: plantlets with 5–7 branch crowns, approximately 4 weeks after subculture; P5: plantlets with over 10 branch crowns, approximately 6 weeks after subculture) were harvested. *Arabidopsis thaliana* ecotype Col-gl was grown at 22°C with 75% relative humidity under short-day (8 h of light at 125 μmol⋅m^-2^⋅s^-1^ and 16 h of dark) conditions for 4–5 weeks before transformation.

Six-month-old uniformly developed strawberry plantlets were selected for the stress treatments. Salt stress was simulated by irrigating potted strawberry plants with 300 mM NaCl. Another set of control ‘Heilongjiang-3’ plantlets was similarly treated with distilled water. Cold and heat stress treatments were performed by transferring the plants to a 4°C/42°C chamber for 48 h. Another set of potted ‘Heilongjiang-3’ plantlets was maintained in the control temperature range from 22 to 27°C. Hormone treatments were performed by spraying the strawberry leaves with a solution containing 0.1 mM abscisic acid (ABA), 1 mM salicylic acid (SA), 0.1 mM methyl jasmonate (MeJA), or 0.5 g/L ethephon (Eth), while another set of control ‘Heilongjiang-3’ plantlets were similarly sprayed with distilled water. The leaves of all of the above plants treated with salt, cold, heat, and hormone stresses were then collected at 0, 0.5, 2, 4, 8, 12, 24, and 48 h post-treatment (hpt). Drought stress treatment was performed by withholding water, followed by sampling at 0, 24, 48, 72, 96, 120, and 144 hpt. The plants were watered again after 144 h of drought stress and sampled after another 24 h. Strawberry plantlets grown without drought stress were used as a control. The powdery mildew (*Podosphaera aphanis*) treatment experiment was conducted by touching the adaxial epidermis with sporulating colonies on the surface of the strawberry leaves. The inoculated leaves were collected at 0, 24, 48, 72, 96, 120, 144, and 168 h post-inoculation (hpi), and uninfected leaves served as a negative control. At each time point of each treatment, six leaves from six separate plants were combined to form one sample, and all of the treatments were evaluated in triplicate. All of the collected plant samples were immediately frozen in liquid nitrogen and stored at –80°C until use.

### Identification of Strawberry *TCP* Genes

To identify *TCP* genes in strawberry, we downloaded the full-length amino acid sequences of hypothetical TCP transcription factor in the diploid woodland strawberry (*F. vesca*) accession Hawaii-4 from the Plant Transcription Factor Database^1^. Next, the full-length amino acid sequences of the hypothetical TCP proteins were employed as query to perform BLAST-P searches in National Center for Biotechnology Information (NCBI) database^2^. An E-value of 1*e*-10 was used as the threshold to ensure the discovery of all potential TCP DNA binding domain-encoding sequences in the strawberry genome (*F. vesca* Annotation Release 101). Finally, the putative *TCP* genes were manually verified using the InterProScan program^3^ and Pfam database^4^ to validate the presence and completeness of a TCP DNA-binding domain (PF03634). The identified strawberry *TCP* genes were annotated based on their respective linkage group distribution. Linkage group assignments were retrieved from annotations downloaded from the NCBI Map Viewer^5^. *FvTCP* genes were mapped to the linkage groups using MapInspect software. The TCP protein sequences from *Arabidopsis thaliana*, apple, rice, and tomato were retrieved from the Plant Transcription Factor Database (PlantTFDB^6^).

### Multiple Sequence Alignments and Phylogenetic Analysis

Multiple sequence alignments of TCP proteins and domains were performed separately using ClustalX 2.0.12, and the alignment results were presented and manually modified using GeneDoc. Target prediction for miR319 was performed using the psRNATarget online tool^7^. The full-length amino acid sequences of the putative TCP proteins from *Arabidopsis* (*AtTCP*), apple (*MdTCP*), strawberry (*FvTCP*), tomato (*SlTCP*), and rice (*OsTCP*) were used to generate a phylogenetic tree based on MUSCLE alignment and the unrooted neighbor-joining method with MEGA 5.0. Neighbor-joining analysis with pairwise deletion and bootstrap analysis with 1000 replicates was performed using the p-distance model (Wei et al., 2016).

### Conserved Motif Identification and Gene Structure Analysis

TCP protein sequences in *F. vesca* were submitted to the online Multiple Expectation maximization for Motif Elicitation (MEME^8^) program to identify conserved protein motifs. The optimized MEME parameters were as follows: minimum motif width, 6; maximum motif width, 100; and maximum number of motifs, 12. The *FvTCP* genomic sequences and CDS sequences extracted from NCBI were compared using the online program GSDS 2.0^9^ to infer the exon/intron organization of the *FvTCP* genes.

### Putative Promoter *cis*-Acting Element Analysis

*FvTCP* nucleotide sequences were used to retrieve whole genomic sequences from NCBI using their gene IDs^10^. The upstream 1 kb region of the translation start site of the *FvTCP* genes was used for putative promoter *cis*-acting element analysis in PlantCARE^11^. The motifs putatively involved in plant growth and development, phytohormone responses, and abiotic and biotic stress responses are summarized.

### Gene Transcript Analysis

To describe the transcript accumulation profiles of *TCP* genes in *F. vesca*, total RNA was extracted from tissue samples or treated leaves using an EZNA Plant RNA Kit (R6827-01, Omega Bio-tek, USA), according to the manufacturer’s protocol. Prior to reverse-transcription, RNA was treated with DNase I (RNase free; TaKaRa Biotechnology, Dalian, China) to remove any residual contaminating genomic DNA. Next, 1.5 μg of total RNA isolated from each sample was reverse-transcribed into first-strand cDNA using PrimeScript RTase (TaKaRa Biotechnology, Dalian, China). Gene-specific primers for each *FvTCP* gene were designed using VECTOR NTI.

Semi-quantitative reverse-transcription (RT) PCR reactions were conducted using the following profile: 95°C for 3 min, 29–33 cycles of 95°C for 30 s, 55°C for 30 s and 72°C for 30 s, and a final step at 72°C for 5 min. The PCR products were separated in a 1.5% (w/v) agarose gel, stained with ethidium bromide, and imaged under UV light for further gene transcript analysis. Each reaction was performed in triplicate, with three independent analyses for each treatment showing the same trends for each gene. The transcript profiles obtained by semi-quantitative RT-PCR were collated and analyzed using GeneSnap software. The heat maps and hierarchical clustering of gene transcript data were visualized in Multi Experiment Viewer (MeV) 4.9 software (Saeed et al., 2006; Guo et al., 2014). Reverse transcription quantitative PCR (RT-qPCR) was performed as described previously with some modifications (Wei et al., 2016). We performed RT-qPCR in a 21-μl reaction volume containing 10.5 μl of SYBR Green Premix Ex Taq II (TaKaRa Biotechnology), 1.0 μl of cDNA, 0.8 μl of 1.0 μM of each primer, and 7.4 μl of sterile distilled H_2_O, using an IQ5 real time-PCR machine (Bio-Rad, Hercules, CA, USA). RT-qPCR amplification was performed under the following conditions: 40 cycles at 95°C for 30 s and 58°C for 30 s. After amplification, the samples were maintained at 50°C for 1 min, and the temperature was gradually increased by 0.5°C every 10 s to perform the melting curve analysis. The *interspacer 26S-18S* strawberry RNA gene (housekeeping gene; *Fv18S*) was used as an internal control to normalize the expression data (Raab et al., 2006; Hu et al., 2015; Medina-Puche et al., 2015; Paniagua et al., 2016; Wei et al., 2016). Three biological replicates with three technical replicates were assayed for each sample. Reactions for the reference gene were included in each plate. The relative transcript levels of the genes were calculated using the 2^-ΔΔCt^ method, and the standard deviation was calculated from three biological replicates. The gene-specific primers are listed in Supplemental Table S1.

### Subcellular Localization of Strawberry *TCP* Genes

Based on the public NCBI database (*F. vesca* Annotation Release 101; Darwish et al., 2015), we designed 10 pairs of gene-specific primers to clone the randomly selected 10 *TCP* genes in the diploid woodland strawberry *F. vesca* accession Heilongjiang-3. The predicted coding sequences of the selected *FvTCP* genes were amplified with high-fidelity HS polymerase (TaKaRa Biotechnology, Dalian, China) using the primers listed in Supplementary Table S1. Finally, six *FvTCP* genes (*FvTCP7*, *FvTCP8*, *FvTCP9*, *FvTCP13*, *FvTCP15*, and *FvTCP17*) belonging to different types were successfully cloned from the cDNA of the leaves of ‘Heilongjiang-3’.

To construct GFP-tagged FvTCP, the six cloned *FvTCP* sequence-encoding genes were cloned into the *Xba*I and *Kpn*I sites in the pBI221 vector. The plasmid DNA of pBI221 containing *35S::FvTCPs-GFP* was introduced into *Arabidopsis* mesophyll protoplasts using the PEG-Ca^2+^-mediated transformation method (Yoo et al., 2007; Wei et al., 2016). The localization of the fusion protein was observed using an Olympus BX-51 inverted fluorescence microscope (Olympus, Japan). GFP fluorescence, the bright field image, and the red autofluorescence of chloroplasts from the protoplast expression assay were imaged simultaneously and merged together. All transient expression assays were repeated at least three times.

### Transfection of Strawberry Fruits by Agroinfiltration and Western Blot Analysis

To produce a YFP-tagged FvTCP9, the coding sequence of *FvTCP9* was cloned in-frame into the *Bam*HI sites of the binary expression vector C15 to generate plasmid *35S::FvTCP9-YFP*. The recombinant plasmids *35S::FvTCP9-YFP* and *35S::YFP* (C15 empty vector) were transfected into *Agrobacterium tumefaciens* strain GV3101 via electroporation. C15 empty vector and *Agrobacterium tumefaciens* strain GV3101 served as the control. *Agrobacterium*-mediated transient assays were performed as described previously with some modifications (Han et al., 2015). The *Agrobacterium* suspension was evenly injected throughout the entire *Fragaria* × *ananassa* Duch. ‘Toyonoka’ strawberry fruit while it was still attached to the plant, at approximately 12 days after anthesis, using a sterile 1-ml hypodermic syringe. The fruits were harvested for Western blot analysis and ripening-related genes transcript analysis at 3 days after infiltration, and the receptacles were frozen in liquid nitrogen and stored at –80°C until use. For each construct, five uniformly sized fruits were used in the infiltration experiment, and the experiment was repeated three times. The primers used for RT-qPCR analysis of ripening-related genes are listed in Supplemental Table S3 (Han et al., 2015).

For Western blot analysis of FvTCP9 protein, total protein was extracted from infiltrated fruits as described previously with some modifications (Wang et al., 2006). The protein was fractionated by 12% SDS-PAGE and blotted onto PVDF membranes (Millipore) using a semi-dry blotting apparatus as described by the manufacturer (Bio-Rad). GFP was detected using a polyclonal mouse anti-GFP antibody (1:5000 dilution; TransGen Biotech, Beijing, China) and a goat anti-mouse IgG secondary antibody (1:10000 dilution; TransGen Biotech, Beijing, China), according to the manufacturer’s instructions. Proteins separated by SDS-PAGE were detected using the SuperSignal West Pico Chemiluminescent Substrate (Thermo Scientific, Rockford, IL, USA).

### Statistical Analysis

Statistical significance was determined using a paired Student’s *t*-test^12^. The mean ± standard deviations from the mean (SD) of at least three replicates are presented, and significant differences relative to controls are indicated at ^∗∗^*p* < 0.05 and ^∗^*p* < 0.01.

## Results

### Identification and Cloning of Strawberry *TCP* Genes

A total of 21 strawberry *TCP* members were originally obtained via a BLAST-P search in NCBI using the 18 strawberry TCP amino acid sequences that were predicted in the sequenced diploid woodland strawberry genome accession Hawaii-4^13^ (Shulaev et al., 2011; Darwish et al., 2015). Subsequently, to verify the reliability of the initial results, a survey was conducted to confirm the presence of the conserved TCP domain using the InterProScan database^14^ and the Pfam database^15^. The results showed that two putative strawberry *TCP* genes (XP_011467829.1 and XP_004298742.1) were discarded because they lacked a conserved TCP domain. As a result, 19 non-redundant *FvTCP* genes were identified in the diploid woodland strawberry and further annotated as *FvTCP1* to *FvTCP19* according to their locations in strawberry linkage groups 1–7 (**Table 1**). The 19 *FvTCP* genes were unevenly distributed in seven linkage groups, with five *FvTCP* genes located linkage groups 5 and 6, while none of the *FvTCP* genes mapped to linkage group 1 (**Supplementary Figure S1**). Detailed characteristics of the TCP transcription factors in *F. vesca* are provided in **Table 1**.

**Table 1:** Characteristics of strawberry *TCP* genes.

Gene name	Gene ID	Accession no.	CDS (bp)	Deduced polypeptide			Chromosomes Location	Type
				No. of aa	pI	Mw (Da)		
FvTCP1	105350143	XP_011459878.1	480	159	7.79	17553.61	LG2: 26038399–26038878	PCF
FvTCP2	105350144	XP_011459879.1	528	175	4.62	18747.64	LG2: 26056242–26056769	PCF
FvTCP3	101296182	XP_004293547.1	1383	460	7.49	49952.47	LG3: 2705162–2712015	CIN
FvTCP4	105350788	XP_011462025.1	696	231	5.29	25832.46	LG3: 7198665–7199360	CIN
FvTCP5	101298687	XP_004294116.1	1320	439	6.69	47786.10	LG3: 10262636–10265179	CIN
FvTCP6	101314849	XP_004298309.1	1245	414	9.55	46760.15	LG4: 16353180–16355880	CYC/TB1
FvTCP7	101307686	XP_004297311.1	1266	421	6.91	44592.24	LG4: 18062660–18064607	PCF
FvTCP8	101296009	XP_004300750.1	1170	389	8.67	40431.04	LG5: 800234–802402	PCF
FvTCP9	101312450	XP_004301078.1	1356	451	6.47	50831.19	LG5: 6995443–6997610	CYC/TB1
FvTCP10	101297843	XP_004301109.2	1131	376	8.82	41441.07	LG5: 7568330–7570484	CIN
FvTCP11	101310025	XP_004299376.1	1272	423	6.74	45402.68	LG5: 8357566–8359447	PCF
FvTCP12	101303815	XP_004301414.1	855	284	9.25	29893.08	LG5: 13952178–13953577	PCF
FvTCP13	101301310	XP_004303161.1	1101	366	6.38	40307.13	LG6: 15460767–15463165	CIN
FvTCP14	105352424	XP_011467828.1	816	271	9.32	30672.82	LG6: 30275770–30276834	CYC/TB1
FvTCP15	101292524	XP_004304223.1	1101	366	6.37	40186.12	LG6: 30960965–30963361	CIN
FvTCP16	105352516	XP_011468122.1	792	263	9.12	28678.13	LG6: 34978600–34980774	PCF
FvTCP17	101293616	XP_011468196.1	1002	333	8.97	35933.53	LG6: 36341802–36343999	PCF
FvTCP18	101309200	XP_004308417.1	870	289	9.51	30862.96	LG7: 6073756–6075554	PCF
FvTCP19	101301345	XP_004309238.2	984	327	6.66	34335.69	LG7: 22954998–22956532	PCF

Based on the predicted *FvTCP* coding sequences, we originally cloned the randomly selected 10 homologous *TCP* genes in the diploid woodland strawberry *F. vesca* accession Heilongjiang-3. Finally, six *FvTCP* genes (*FvTCP7*, *FvTCP8*, *FvTCP9*, *FvTCP13*, *FvTCP15*, and *FvTCP17*) belonging to different types were successfully cloned from the cDNA of the leaves of the *F. vesca* accession Heilongjiang-3 and submitted to NCBI GenBank (GenBank Accession Numbers KX227709, KX227710, KX227711, KX227712, KX227713, and KX227714, respectively). The CDS sequences of the six cloned *FvTCP* genes from accession Heilongjiang-3 also shared high identities (≥99%, *E*-value = 0, data not shown) with the corresponding *FvTCPs* in the strawberry accession Hawaii-4.

### Phylogenetic Analysis and Classification of Strawberry *TCP* Genes

**FIGURE 1:**
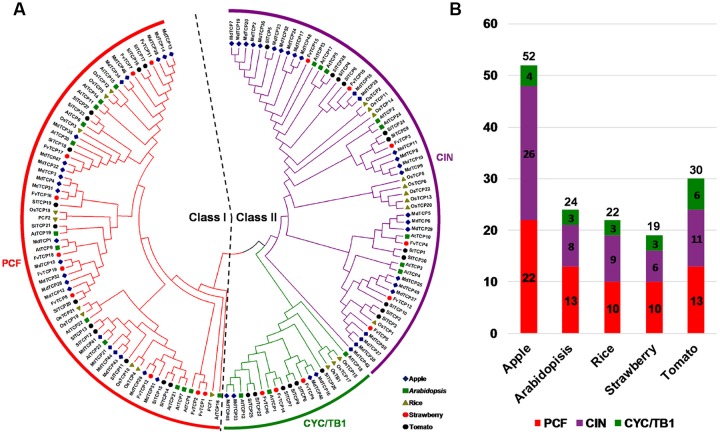
**Phylogenetic analysis of *TCP* proteins from strawberry, *Arabidopsis*, apple, rice, and tomato. (A)** The full-length amino acid sequences of *TCP* proteins from strawberry (FvTCP), *Arabidopsis* (AtTCP), apple (MdTCP), rice (OsTCP), and tomato (SlTCP) were aligned by ClustalX, and the phylogenetic tree was constructed using the neighbor-joining method with 1000 bootstrap replicates by MEGA 5.0. The branched lines of the subtrees are colored to indicate different TCP subgroups. **(B)** TCP family members of apple, *Arabidopsis*, rice, strawberry, and tomato.

**FIGURE 2:**
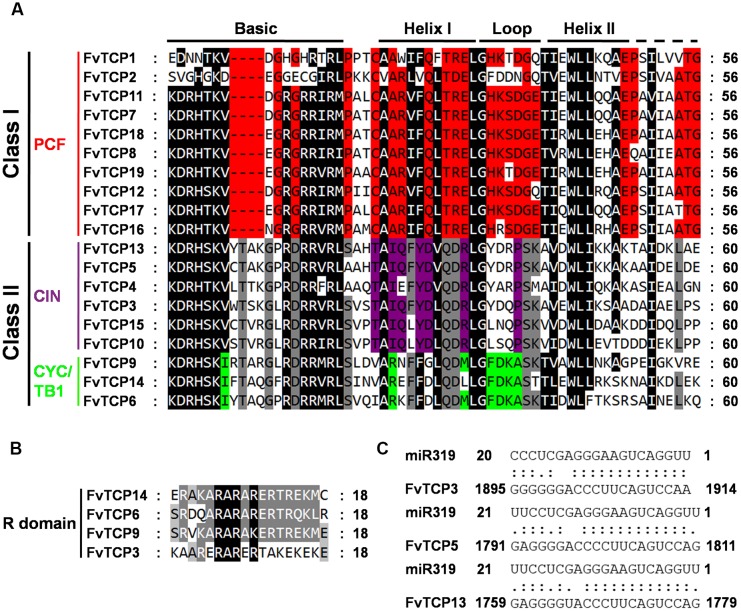
**Multiple sequence alignment of strawberry TCP transcription factors. (A)** Alignment of the TCP domain for the predicted strawberry TCP proteins. Black boxes highlight residues conserved in both TCP classes; red, residues conserved in class I; gray, conserved in class II; purple, conserved in CIN-like proteins; light-green, conserved in CYC/TB1 proteins. The basic, helix I, loop, and helix II regions are indicated. **(B)** Alignment of the R-domain of Class II subfamily members. The sequences were aligned with ClustalW and visualized with Genedoc. **(C)** Alignment of putative target areas for miR319.

To assess the classification of the *FvTCP* genes and to gain some insight into the potential function of FvTCP proteins from well-studied TCPs in other plant species, a total of 147 *TCP* genes, comprising 24 genes from *Arabidopsis*, 52 from apple, 22 from rice, 19 from strawberry and 30 from tomato, were used to construct a phylogenetic tree (**Figure 1**). Additionally, to gain a better understanding of the classification of *FvTCP* members, multiple sequence alignment was performed spanning the approximately 60-amino-acids core TCP domain of all FvTCP amino acid sequences. The phylogenetic analysis and the TCP domain alignment showed that strawberry TCP proteins can be divided into two classes: class I (or PCF) and class II (**Figure 1A**). The TCP proteins of all five plant species were classified into two major classes (class I and class II; **Figure 1B**). The most striking difference between these two classes was a four-amino-acid deletion in the basic domain of class I relative to class II proteins (**Figure 2A**). Further analysis showed that the phylogenetic tree also supported the *Arabidopsis* and rice described previously in the classification of class II proteins in CIN and CYC/TB1 two subclades (Yao et al., 2007). According to this classification, the CYC/TB1 subclade contains three *FvTCP* genes (*FvTCP6*, *FvTCP9*, and *FvTCP14*), and the CIN subclade contains six *FvTCP* genes (*FvTCP3*, *FvTCP4*, *FvTCP5*, *FvTCP10*, *FvTCP13*, and *FvTCP15*; **Figure 2A**). Outside the TCP domain, the R domain, an approximately 18-residues arginine-rich motif, is conserved and only present in a subset of class II proteins. As shown in **Figure 2B**, four class II genes (*FvTCP6*, *FvTCP9*, *FvTCP14*, and *FvTCP3*) contain the R domain, but *FvTCP3* is less conserved. Additionally, three CIN subclade genes (*FvTCP3*, *FvTCP5*, and *FvTCP13*) contained the putative miR319 target site and shared the highest sequence similarity with the *Arabidopsis* and tomato miR319-targeted *TCP* genes (**Figures 1A** and **2C**).

### Conserved Motif Identification and Gene Structure Analysis

**FIGURE 3:**
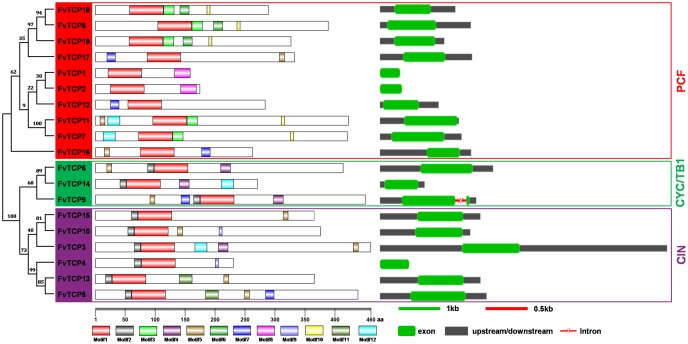
**Structural analysis of strawberry TCP transcription factors.** The protein domains of the strawberry *TCP* genes are shown on the left and are denoted by rectangles with different colors. The exon-intron organization is shown on the right, with exons and introns represented by green round-corner rectangles and red shrunken lines, respectively; untranslated regions (UTRs) are indicated by gray rectangles. The red, green, and purple rectangles are used to cluster the genes into the PCF, CYC/TB1, and CIN classes.

To obtain a better understanding of the diversification and evolutionary relationships of the TCP protein in *F. vesca*, the conserved motifs and exon/intron organization of FvTCPs were analyzed. A new phylogenetic tree was constructed using the protein sequences of FvTCPs, which divided the FvTCP proteins into three subclades. As shown in **Figure 3** and **Supplementary Figure S2**, we used the online MEME tool to predict the conserved FvTCP protein motifs, identifying 12 conserved motifs. As expected, all 19 of the FvTCPs demonstrated the presence of a highly conserved TCP domain (motif 1). The conserved R domain (motif 4) was found in four class II FvTCPs. All of the class II FvTCPs were characterized by motif 2 in the N-terminal TCP domain. By comparison, the C-terminal TCP domain of motif 3 was detected in a subset of the class I proteins. In addition, some motifs were exclusively present in a subset of a particular subclade, suggesting that these motifs may contribute to the specific function of those genes in the subclade. For example, motifs 6, 8, and 10 in PCF, and motifs 9 and 11 in CIN (**Figure 3**).

To gain further insight into the evolutionary relationships among *FvTCP* genes, we investigated the exon/intron organization of individual *FvTCP* genes by aligning the cDNA sequences and corresponding genomic DNA sequences. Overall, the *FvTCP* genes exhibited a highly conserved exon-intron organization: 18 of 19 *FvTCP* genes had no intron, while only *FvTCP9* genes possessed one intron. Additionally, most of the *FvTCP* genes within the nearby paralogous genes demonstrated very similar exon/intron distribution patterns in terms of the exon length and the intron number (**Figure 3**).

### Identification of *cis*-Regulatory Elements in the Promoter of *FvTCP* Genes

**FIGURE 4:**
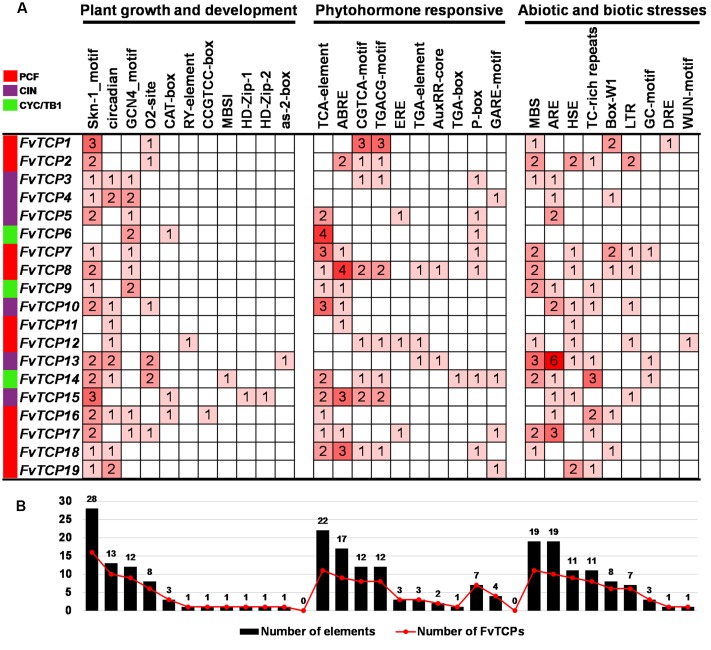
***Cis*-acting element analysis of the promoter regions of strawberry *TCP* genes. (A)** Number of each *cis*-acting element in the promoter region (1 kb upstream of the translation start site) of *FvTCP* genes. **(B)** Statistics for the total number of *FvTCP* genes, including the corresponding *cis*-acting elements (red dot) and the total number of *cis*-acting elements in *FvTCP* gene family (black box). Based on the functional annotation, the *cis*-acting elements were classified into three major classes: plant growth and development-, phytohormone responsive-, or abiotic and biotic stresses-related *cis*-acting elements (detailed results shown in Supplementary Table S2).

The analysis of *cis*-regulatory elements in promoter sequences is an important feature for understanding gene function and regulation. To identify the likely *cis*-acting elements of the *FvTCPs*, the promoter regions (1 kb of genomic DNA sequence upstream of the translation start site) of the *FvTCP* genes were used to search the PlantCARE database (Postel et al., 2002). A series of *cis*-acting elements involved in plant growth and development, phytohormone responses, and abiotic and biotic stress responses were identified (**Supplementary Table S2**). As shown in **Figure 4**, the Skn-1_motif and GCN4_motifs, *cis*-acting regulatory elements involved in endosperm expression (Washida et al., 1999), were found in the promoters of 16 and 9 *FvTCP* genes, respectively. The circadian control element (circadian; Anderson et al., 1994) was found in 10 *FvTCP* genes. Notably, two leaf development-related *cis*-acting elements (HD-Zip1 and HD-Zip2; Sessa et al., 1993) were found in the *FvTCP15* promoter. Additionally, the zein metabolism regulation element (O2 site), meristem expression and specific activation element (CAT-box and CCGTCC-box), seed-specific regulation element and shoot-specific expression element (RY element and as-2-box; Bobb et al., 1997) were also identified in the promoters of the *FvTCP* genes. In hormone-related *cis*-acting elements, the SA-responsive element (TCA element; Goldsbrough et al., 1993), the MeJA-responsive element (CGTCA motif and TGACG motif; Rouster et al., 1997), and the ABA-responsive element (ABRE; Shen and Ho, 1995) were found in the promoters of 11, 8, and 9 *FvTCP* genes, respectively. The gibberellin-responsive element (GARE motif and P-box; Kim et al., 1992; Washida et al., 1999) and the auxin-responsive element (TGA element, AuxRR core and TGA box; Ulmasov et al., 1997) were observed in 10 and 4 *FvTCP* genes. Plenty of hormone-responsive elements were identified in the *FvTCP* promoter sequences, indicating that phytohormones could play crucial roles in the regulation of plant growth and development (**Figure 4B**). In addition, some stresses-related (e.g., drought, extreme temperatures, salinity, and disease) *cis*-acting elements were also found in the putative promoter regions of the *FvTCP* genes (**Figure 4**).

### Tissue-Specific Transcript Accumulation Patterns in *FvTCP* Genes

**FIGURE 5:**
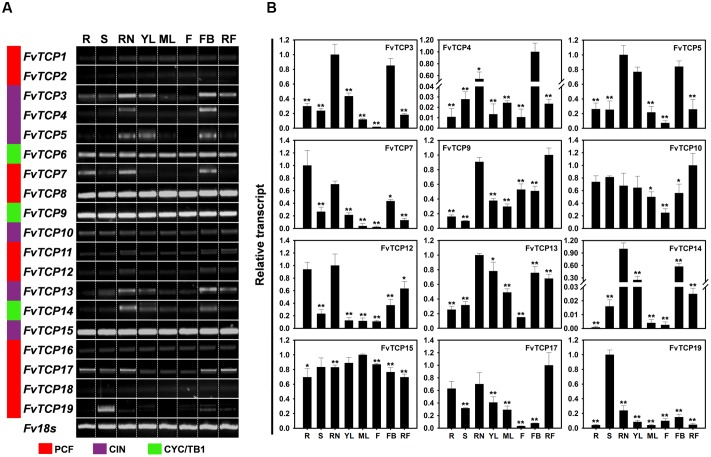
**Tissue-specific transcript accumulation patterns of 19 *TCP* genes in the diploid woodland strawberry (*F. vesca*). (A)** Transcript accumulation profiles of 19 *FvTCP* genes in different tissues using semi-quantitative PCR. *Fv18s* was used as an internal control. Lanes: R, roots; S, stems; RN, runners; YL, young leaves; ML, mature leaves; F, flowers; FB, floral buds; RF, red fruits. **(B)** Transcript accumulation profiles of 12 selected *FvTCP* genes in different tissues using RT-qPCR. The analysis results were normalized using *Fv18s*. The experiments were repeated three times and provided consistent results. The mean values and SDs were obtained from three biological and three technical replicates. Asterisks indicate significant difference compared to the highest transcription level, as determined by Student’s *t*-test (^∗^*p* < 0.05, ^∗∗^*p* < 0.01).

To investigate the tissue-specific transcript accumulation profiles of *TCP* genes in *F. vesca*, we analyzed the transcripts of *FvTCP* genes using semi-quantitative RT-PCR and validated the results via RT-qPCR analysis of different tissues, including roots, stems, runners, leaves, flowers, floral buds, and fruits (fully ripened fruits) from the diploid woodland strawberry accession Heilongjiang-3. As indicated in **Figure 5**, some *FvTCP* genes exhibited tissue-specific transcript accumulation patterns, while other *FvTCP* genes showed similar transcript accumulation patterns in different tissues, potentially indicating the functional divergence of *FvTCP* genes during strawberry growth and development. For example, *FvTCP6*, *FvTCP8*, *FvTCP9*, and *FvTCP15* were constitutively expressed in every tissue tested at relatively high transcript levels, whereas *FvTCP1*, *FvTCP2*, and *FvTCP18* were expressed at very low level in all tested tissues (**Figure 5A**). In contrast, the transcript accumulation levels of *FvTCP3* and *FvTCP5* were very high in runners, young leaves, and floral buds and they were relatively low in flowers, indicating that they might play an important role in the development of runners, young leaves, and floral buds. A similar transcript accumulation pattern was observed for *FvTCP13* (**Figure 5B**). In particular, *FvTCP19* displayed extremely high relative transcript levels in stems, which suggested that it might play a role in the development of strawberry stems. *FvTCP4* and *FvTCP14* were preferentially expressed at high levels in runners, young leaves, or floral buds and at almost undetectable levels in other tissues (**Figure 5B**). Additionally, the transcript accumulation patterns of certain genes varied among the eight tissues. For example, *FvTCP12* and *17* exhibited high transcript levels in roots, runners, and fruits, but relatively low transcript levels in young leaves, mature leaves, flowers, and floral buds, and *FvTCP7*, which was abundantly expressed in roots, runners, and floral buds, displayed lower levels in other tissues (**Figure 5B**). Remarkably, RT-qPCR analysis showed that some *FvTCP* genes (*FvTCP9*, *FvTCP10*, and *FvTCP17*) were highly expressed in fruits (fully ripened fruits), which indicated that they might play an important role in fruit ripening (**Figure 5B**). These results prompted us to investigate the transcript of *FvTCP* genes during various fruit development and ripening stages.

### Transcript Accumulation Patterns of *FvTCP* Genes during Different Fruit Developmental Stages

**FIGURE 6:**
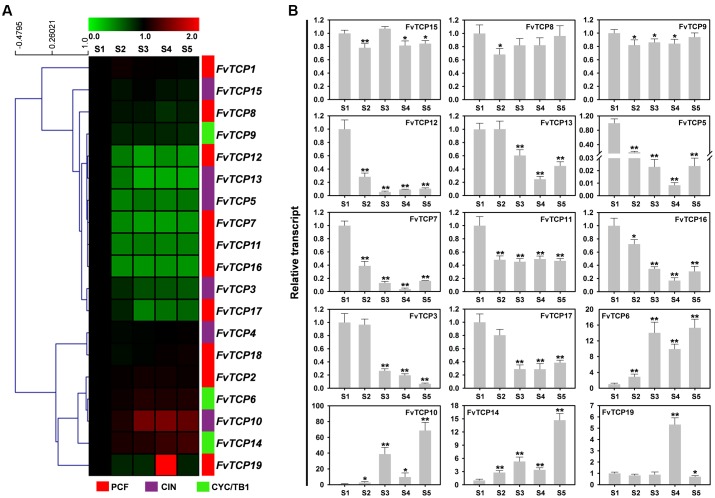
**Transcript accumulation pattern of 19 *TCP* genes in the diploid woodland strawberry (*F. vesca*) during different fruit developmental stages. (A)** Hierarchical clustering of the transcript accumulation profiles of 19 *FvTCP* genes during different fruit developmental stages (S1: mature flowers with partially withered petals, S2: mature green receptacles, S3: white receptacles with green achenes, S4: half white and half red fruits, S5: fully ripened fruits; original results shown in **Supplementary Figure S4**). *Fv18s* was used as an internal control. The transcript accumulation profiles were generated by semi-quantitative PCR and were visualized as heat maps. The color scale represents the relative transcript levels with increased (red) or decreased (green) transcript abundance. Genes were hierarchically clustered based on average Pearson’s distance metric and ‘average linkage’ method. **(B)** RT-qPCR transcript analysis of 15 selected *FvTCP* genes during different fruit developmental stages. The results were normalized to *Fv18s*. The experiments were repeated three times and provided consistent results. The mean values and SDs were obtained from three biological and three technical replicates. Asterisks indicate significant difference compared to S1, as determined by Student’s *t*-test (^∗^*p* < 0.05, ^∗∗^*p* < 0.01).

To investigate fruit development and ripening-related strawberry *FvTCP* genes, we focused on the transcript accumulation patterns of *FvTCP* genes in fruits during five different developmental stages (mature flowers with partially withered petals, mature green receptacles, white receptacles with green achenes, half white and half red fruits, and fully ripened fruits). Hierarchical clustering was used to describe the various relative levels of *FvTCP* gene transcripts, which could be differentiated into two distinct groups. As shown in **Figure 6A**, 11 *FvTCP* genes were down-regulated during different fruit developmental stages, while five *FvTCP* genes exhibited up-regulated transcript accumulation patterns. Additionally, three *FvTCP* genes showed stable transcript accumulation patterns. Among the 16 down-regulated and up-regulated *FvTCP* genes, we selected 15 relatively dramatically down-regulated or up-regulated *FvTCP* genes by RT-qPCR to further test their transcript abundance in the various developmental stages of the fruit (**Figure 6B**). Among them, five *TCP* genes (*FvTCP5*, *FvTCP7*, *FvTCP11*, *FvTCP12*, and *FvTCP16*) exhibited high transcript accumulation patterns in mature flowers with partially withered petals, and three *TCP* genes (*FvTCP3*, *FvTCP13*, and *FvTCP17*) showed high accumulation patterns in mature flowers with partially withered petals and mature green receptacles in comparison with the other stages (**Figure 6B**). These data indicate that the eight *FvTCP* genes may be involved in strawberry fruit development. The transcripts of four genes (*FvTCP6*, *FvTCP10*, *FvTCP14*, and *FvTCP19*) were gradually upregulated during the ripening process, which suggested that they might function in strawberry fruit ripening (**Figure 6B**).

### Transcript Accumulation Patterns of *FvTCP* Genes during Different Strawberry Subcultural Propagation Periods

To provide additional information on the growth and developmental functions of *TCP* genes in strawberry, we investigated their transcript accumulation patterns during five different periods of subcultural propagation in strawberry ‘Heilongjiang-3’ using semi-quantitative RT-PCR (**Supplementary Figure S3A**). In general, the transcript accumulation patterns obtained for the *FvTCP* genes could be classified into two types (**Supplementary Figure S3B**). The majority of the *FvTCP* genes, specifically *FvTCP2*, *FvTCP4*, *FvTCP6*, *FvTCP10*, *FvTCP11*, *FvTCP12*, and *FvTCP14*, exhibited downregulation from P1 to P5 stage. The remaining *FvTCP* genes were only upregulated during specific propagation periods. For example, *FvTCP8*, *FvTCP9*, *FvTCP13*, *FvTCP18*, and *FvTCP19* were upregulated at P2 stage, which indicated that they might promote growth and development during early subcultural propagation. Additionally, the transcript levels of *FvTCP1*, *FvTCP9*, and *FvTCP18* revealed an upregulation at P5 stage, suggesting that these genes might be involved in rooting and bud germination after subculture. *FvTCP17* displayed high transcript levels from P2 to P4 stage, implying that *FvTCP17* might play vital biological roles in the subcultural propagation processes of whole strawberry.

### Transcript Analysis of *FvTCP* Genes in Response to Stresses and Hormone Treatments

**FIGURE 7:**
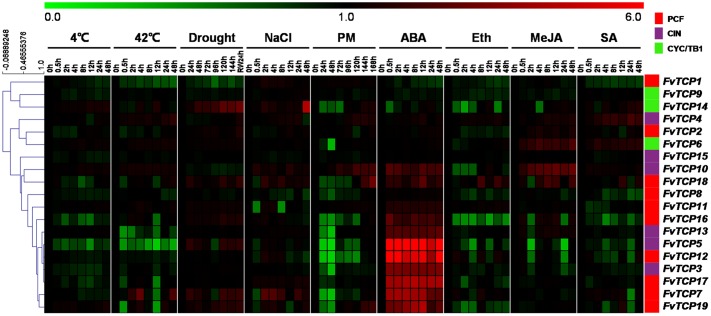
**Hierarchical clustering of the transcript accumulation profiles of 19 *TCP* genes in the diploid woodland strawberry (*F. vesca*) in response to different treatments.** The transcript accumulation profiles of 19 *FvTCP* genes in response to cold, heat, drought, NaCl, powdery mildew infection, ABA, Eth, MeJA, and SA treatments were generated by semi-quantitative PCR and visualized as heat maps (original results shown in **Supplementary Figures S5** and **S6**). The color scale represents the relative transcript levels with increased (red) or decreased (green) transcript abundance. Genes were hierarchically clustered based on average Pearson’s distance metric and ‘average linkage’ method. The experiments were repeated three times with consistent results.

To determine the potential roles of the *FvTCP* genes during plant responses to various environmental stresses, semi-quantitative RT-PCR was performed for the 19 *FvTCP* genes in the leaves of *F. vesca* plantlets exposed to cold, heat, drought, salt, and powdery mildew treatments. Overall, the *FvTCP* genes responded to drought and salt treatment to a greater extent than to temperature and biotic treatment (**Figure 7**). Among them, 10 *FvTCP* genes were upregulated in response to drought stress, and 13 *FvTCP* genes responded to salt treatment. In contrast, a minority of the *FvTCP* genes were only slightly upregulated in response to temperature and biotic treatment (**Figure 7**). Notably, *FvTCP14* and *FvTCP19* showed different degrees of upregulation in response to cold, heat, drought, salt, and powdery mildew treatments. The transcript levels of *FvTCP4*, *7*, and *10* responded to at least three treatments (**Figure 7**).

**FIGURE 8:**
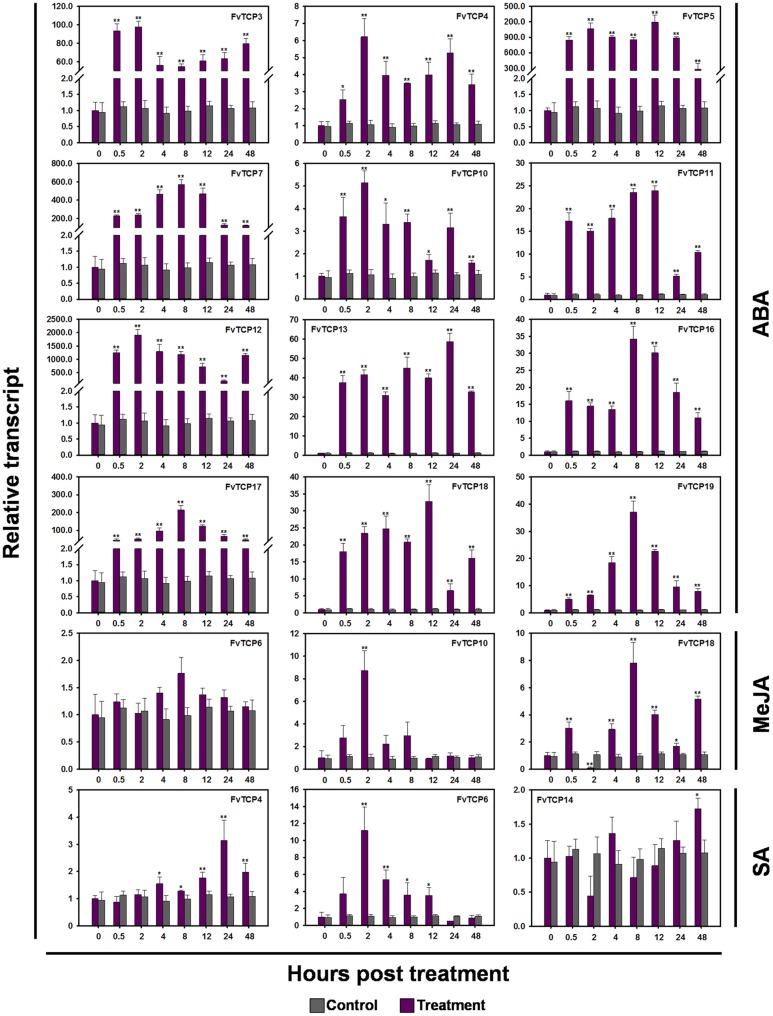
**RT-qPCR analysis of several *TCP* genes in the diploid woodland strawberry (*F. vesca*) in response to ABA, MeJA, and SA treatments.** The detailed transcript levels of several *FvTCP* genes revealed unusual transcript accumulation patterns in response to ABA, MeJA, and SA treatments. The results were normalized to *Fv18s*. The experiments were repeated three times and provided consistent results. The mean values and SDs were obtained from three biological and three technical replicates. The asterisks indicate that the corresponding gene was significantly up or down-regulated in response to treatment, as determined by the Student’s *t*-test (^∗^*p* < 0.05, ^∗∗^*p* < 0.01).

Plant hormones such as SA, MeJA, ethylene (Eth) and ABA have well-established roles in plant stress signaling networks and developmental processes (Bari and Jones, 2009). To understand how *FvTCP* gene transcripts accumulate in response to plant hormone treatment, semi-quantitative RT-PCR and RT-qPCR were used to analyze *FvTCP* transcripts in response to ABA, ethephon (Eth), MeJA and SA in the leaves. For ABA treatment, the transcript levels of 12 *FvTCP* genes were prominent and rapidly increased to significantly high levels at 0.5 hpt, which were maintained throughout the entire treatment period (**Figure 7**). Notably, the highest transcript levels of *FvTCP5*, *7*, *12*, and *17* reached extremely high levels compared with the basal transcript levels after ABA treatment (**Figure 8**). Conversely, almost all *FvTCP* was downregulated or remained nearly unchanged relative to basal transcript levels in response to Eth treatment (**Figure 7**). Additionally, seven and four *FvTCP* genes were upregulated in response to MeJA and SA, respectively, while the other TCP members were downregulated or exhibited no significant change (**Figure 7**). It is worth noting that *FvTCP4*, *FvTCP6*, *FvTCP10*, *FvTCP13*, *FvTCP16*, and *FvTCP18* were upregulated in response to at least two of the hormone treatments (**Figure 7**).

### Subcellular Localization of *FvTCPs*

**FIGURE 9:**
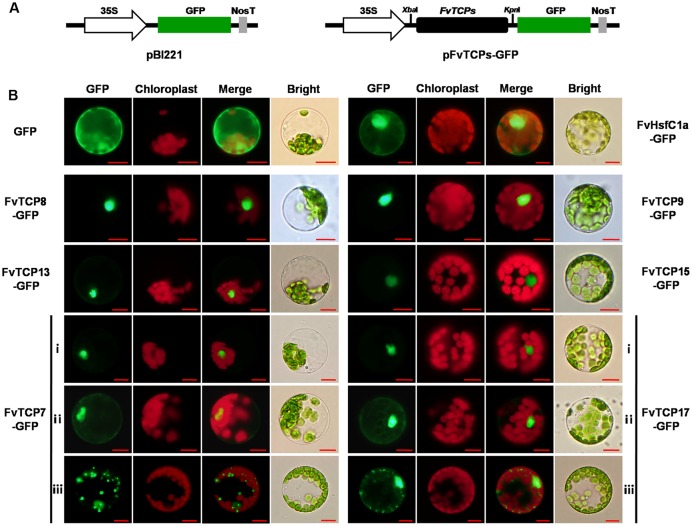
**Subcellular localization of six strawberry *TCP* genes. (A)** Schematic illustration of vectors pBI221 and FvTCPs. The selected *TCP* genes were cloned from a diploid woodland strawberry (*F. vesca*) and used to construct the CaMV35S::TCPs-GFP vectors, in which GFP was fused at the C-terminus. **(B)** The six FvTCP-GFP fusion proteins (FvTCP7-GFP, FvTCP8-GFP, FvTCP9-GFP, FvTCP13-GFP, FvTCP15-GFP, and FvTCP17-GFP), the FvHsfC1a-GFP marker protein, and GFP as a control were transiently expressed in Col-gl *Arabidopsis* protoplasts and observed under a fluorescence microscope. The merged images were constructed in the green fluorescence channel (first panels) and the chloroplast autofluorescence channel (second panels). The corresponding bright field images are shown on the right. *Bar* = 10 μm.

It is known that TFs regulate the transcription of target genes by binding to specific *cis*-elements in their promoters and that this binding occurs in the nucleus. To assess the subcellular localization of the FvTCP TFs, the full-length open reading frames (ORFs) without the stop codon of six cloned *FvTCP* genes were cloned into a vector in-frame with green fluorescence protein (GFP) under the control of the CaMV *35S* promoter. The resulting constructs and empty (control) vector were transiently expressed in *Arabidopsis* mesophyll protoplasts. Fluorescence microscopy revealed that six FvTCP fusion proteins were clearly localized in the nucleus (**Figure 9**). Notably, FvTCP7-GFP (ii) and FvTCP17-GFP (ii) were also localized in the nucleus and cytoplasm (**Figure 9**). To confirm the nuclear and cytoplasm localization of FvTCP7 (ii) and FvTCP17 (ii), we also examined the subcellular localization of FvHsfC1a, a strawberry heat shock transcription factor that has been reported to localize to both the nucleus and cytoplasm of *Arabidopsis* mesophyll protoplasts (Hu et al., 2015). FvTCP7 (ii), FvTCP17 (ii), and FvHsfC1a exhibited similar subcellular localizations (**Figure 9**). Additionally, FvTCP7-GFP (iii) and FvTCP17-GFP (iii) fluorescent signals showed a punctate pattern in the cytoplasm that resembled mitochondria (**Figure 9**).

### Transient Over-Expression of the *FvTCP9* Gene in Strawberry Fruits

**FIGURE 10:**
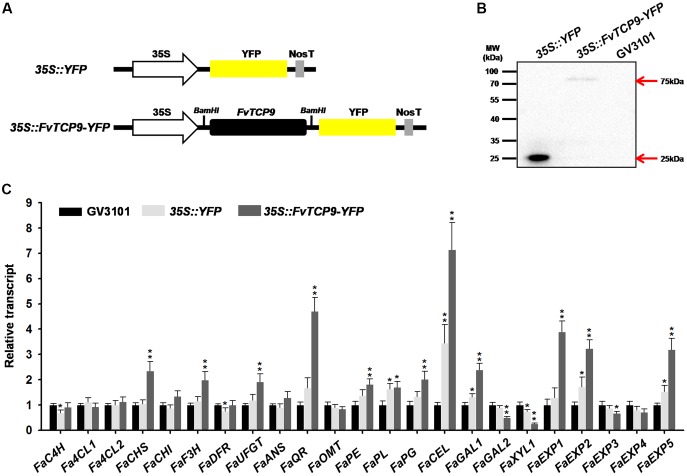
**Transient over-expression of *FvTCP9* in *Fragaria* × *ananassa* Duch. ‘Toyonoka’ fruits. (A)** Schematic illustration of vectors *35S::YFP* and *35S::FvTCP9-YFP*. Black filled boxed denote the *FvTCP9* gene. Yellow filled boxed indicate yellow fluorescence protein (YFP). **(B)** Western blot analysis of FvTCP9 in transiently over-expressing *FvTCP9* strawberry fruits. Samples were harvested at 3 days after agroinfiltration, and total soluble proteins were extracted. **(C)** RT-qPCR transcript analysis of ripening-related genes during the transient over-expression of *FvTCP9* in *Fragaria* × *ananassa* Duch. ‘Toyonoka’ fruits. The strawberry 26S-18S RNA gene (housekeeping gene) was used as an internal control to normalize the expression data. The ripening-related gene primers are listed in **Supplementary Table S3** (Han et al., 2015). Asterisks indicate significant differences compared with the control sample, as determined by the Student’s *t*-test (^∗^*p* < 0.05, ^∗∗^*p* < 0.01).

*Agrobacterium*-mediated transient gene expression is a rapid and powerful tool for the analysis of gene function in plants. For a more detailed analysis of the biological roles of the *FvTCP* genes during strawberry fruit development and ripening, we cloned the *FvTCP9* gene into the *Bam*HI sites of the binary expression vector C15 and transiently over-expressed it in *Fragaria* × *ananassa* Duch. ‘Toyonoka’ attached fruits (12 days after anthesis) (**Figure 10A**). The Western blot results revealed single protein bands of the expected size (∼75 kDa for FvTCP9-YFP and ∼25 kDa for YFP) in the agroinfiltrated strawberry fruits, whereas antigen-specific bands were not detected in protein extracts from agroinfiltrated *Agrobacterium* strain GV3101 control fruits (**Figure 10B**). These results clearly demonstrated the stable integration and protein expression of FvTCP9 in agroinfiltrated strawberry fruits. Additionally, several strawberry fruit ripening-related genes were analyzed by RT-qPCR in the injected fruits. As shown in **Figure 10C**, transient over-expression of *FvTCP9* markedly up-regulated the expression of a series of genes implicated in fruit color and aroma metabolism, including *FaCHS* (chalcone synthase), *FaF3H* (flavanone 3-hydroxylase), *FaUFGT* (UDP-glycose flavonoid 3-O-glycosyltransferase), and *FaQR* (quinine oxidoreductase). Simultaneously, transient over-expression *FvTCP9* dramatically affected the expression of a series of genes implicated in fruit softening, such as *FaPE* (pectinesterase), *FaPG* (polygalacturonase), *FaCEL* (cellulose), *FaGAL1/2* (b-galactosidase1/2), *FaXYL1* (b-xylosidase1), and *FaEXP1/2/5* (expansin1/2/5).

## Discussion

The *TCP* gene family encodes plant-specific transcription factors that are involved in plant growth and development (Manassero et al., 2013). To date, the features and functions of the *TCP* gene family have been identified and investigated in several plant species, including *Arabidopsis* (Riechmann et al., 2000), rice (Yao et al., 2007), tomato (Parapunova et al., 2014), apple (Xu et al., 2014), cotton (Ma et al., 2014), and watermelon (Shi et al., 2016). However, no comprehensive analyses of the TCP gene family in *F. vesca*, an important *Rosaceae* model plant that is widely used in fruit research, have been conducted. In this study, we conducted a broad analysis of the *TCP* genes in strawberry by investigating their linkage group organization, evolutionary relationships, gene structure, protein motifs, *cis*-acting elements, and expression profiles in different tissues and developmental stages and under various stress conditions, subcellular localizations, and during transient over-expression. Genome-wide analysis of the *TCP* genes in *F. vesca* will facilitate a better understanding of the role of this gene family during strawberry growth and development.

### Evolution and Structure of the *TCP* Gene Family in *F. vesca*

A total of 19 *FvTCPs* were identified based on the diploid woodland strawberry genome (accession Hawaii-4; Shulaev et al., 2011; Darwish et al., 2015). This number is highly conserved among *Arabidopsis* (24 members) and rice (22 members; **Figure 1B**) (Riechmann et al., 2000; Yao et al., 2007). However, it is significantly lower than that present in apple (52 members; **Figure 1B**), which is consistent with the genome sizes of apple (∼742.3 Mb in *M. domestica*; Velasco et al., 2010), indicating that TCP genes in different plants have expanded to differing degrees. Sequence alignment and phylogenetic analysis of FvTCP proteins has resulted in their classification into three major subclasses (I, II, and III), with *FvTCP* genes distributed across all three subclasses. In addition, each subclass contains *TCP* genes from *Arabidopsis*, tomato, apple, and rice. *FvTCP* genes are more closely related to genes from apple *TCP* genes, demonstrating that apple and strawberry are *Rosaceae* and evolved more recently from a common ancestor. These results indicate that although plant *TCP* genes may be derived from a common ancestor, many have undergone distinct patterns of differentiation with the divergence of different lineages. Moreover, the consistency of the motif compositions of FvTCP proteins as well as the exon/intron structures of most *FvTCP* genes with phylogenetic subclasses further supported the close evolutionary relationships among *FvTCPs* as well as the dependability of our phylogenetic analysis, as described previously, in cotton and apple (Ma et al., 2014; Xu et al., 2014).

### Potential Roles of *FvTCP* Genes in Plant Growth and Development

Accumulating evidence suggests that TCP transcription factors are involved in the regulation of cell growth and proliferation, performing diverse functions in multiple aspects of plant growth and development (Martin-Trillo and Cubas, 2010). The CYC/TB1 clade includes genes that are mainly involved in the development of axillary meristems that give rise to either flowers or lateral shoots. *AtTCP1*, the closest homolog of *CYC* in *Arabidopsis*, is involved in the longitudinal elongation of petioles, rosette leaves, and inflorescent stems. The expression pattern of *AtTCP1* is strong in the lower part of the inflorescence stem, the distal region of expanding rosette leaves, and the midrib of the blade and petiole during leaf development (Koyama et al., 2010). Additionally, *Arabidopsis* gain-of-function *tcp1-1D* mutant plants show elongated leaves and petioles, whereas *TCP1-SRDX* plants display rounded and epinastic leaves, shortened petioles, and reduced statures (Guo et al., 2010). *FvTCP14*, which is closely related to *AtTCP1*, was transcribed at high levels in runners, floral buds, or young leaves and was almost undetectable in other tissues (**Figures 1A** and **5B**). This result is consistent, in part, with the expression profile of *AtTCP1* and implies that the *FvTCP14* gene in *F. vesca* functions in runners and in inflorescence and leaf development. *BRANCHED1* (*BRC1*, *AtTCP18*) and *BRANCHED2* (*BRC2*, *AtTCP12*), two homologs of *TB1* in *Arabidopsis*, were transcribed at high levels in tissues that mainly contained axillary buds, such as leaf bases and stem, inflorescences, and siliques (Aguilar-Martinez et al., 2007). *AtTCP18* acts downstream of auxin and strigolactone to coordinate axillary bud outgrowth, and mutants with reduced activity of either gene show an increased number of rosette branches. In contrast, the up-regulation of *AtTCP18* results in an inhibition of lateral branching. *AtTCP12* exhibits a weaker or no mutant phenotype compared with *AtTCP18* (Aguilar-Martinez et al., 2007; Finlayson, 2007). The phylogenetically close gene of *AtTCP18* in strawberry is *FvTCP9* (**Figure 1A**), which displays higher homology with *AtTCP18* by NCBI BLAST-P and a significantly higher relative transcript level in runners and fruits (**Figure 5**). These transcript similarities suggest that *FvTCP9* is likely to perform roles similar to *AtTCP18* in axillary bud tissue development in strawberry.

Additionally, CIN-like clade genes could be more ancient than the CYC/TB1 clade TCPs and are important for leaf growth and development. In *Arabidopsis*, five of the CIN subclade members (*AtTCP2*, *3*, *4*, *10*, and *24*) are post-transcriptionally regulated by miRNA319, and miR319 modulates jasmonate biosynthesis, negative leaf curvature, and crinkly leaves, while it positively regulates leaf senescence and affects petal development (Nath et al., 2003; Palatnik et al., 2007; Schommer et al., 2008; Nag et al., 2009). In the present study, the closest strawberry homologs of these *Arabidopsis* genes are the four *FvTCP* genes, *FvTCP3*, *FvTCP4*, *FvTCP5*, and *FvTCP13*, all of which, excluding *FvTCP4*, carry a putative binding site for miR319 (**Figures 1A** and **2C**). The transcript accumulation levels of *FvTCP3*, *FvTCP5*, and *FvTCP13* were very high in runners, young leaves, and floral buds. These results indicated that the regulation of leaf growth and development by miRNA-targeted TCP TFs that are homologous to those in strawberry may be consistent in *Arabidopsis*.

By contrast, Class I *FvTCP* genes showed more widespread and less tissue-specific transcript accumulation patterns, such as in roots, runners, floral buds, and fruits (**Figure 5**). These results implied that Class I *FvTCP* genes might play diverse regulatory roles at multiple growth and development stages. In *Arabidopsis*, *AtTCP14* regulates embryonic growth potential during seed germination, which is related to ABA and GA responses (Tatematsu et al., 2008). In our study, *FvTCP7*, *11*, and *17* were phylogenetically close to *AtTCP14* and had similarly high transcript accumulation levels after ABA treatment (**Figures 1A** and **8**). However, *FvTCP7*, *11*, and *17* were down-regulated during different developmental stages of the fruit (**Figure 6**), suggesting that they were also down-regulated during embryo and seed development. These findings indicate that *FvTCP7*, *11*, and *17* might have functions that differ from those of ABA-mediated embryo and seed development in response to *AtTCP14*.

### *FvTCP* is Likely to Play a Role in Fruit Development and Ripening

Fruit development and ripening is a complex and highly controlled biological process that is controlled by transcriptional regulatory networks involving many transcription factors, such as MADS-box, NAC, and EIN3/EIL (Seymour et al., 2013). In strawberry, the endosperm and seed coat play a crucial role in the fruit set and early stage fruit development (Kang et al., 2013). Promoter analysis showed that most of the *FvTCP* genes harbored Skn-1_motif and GCN4_motif *cis*-regulatory elements involved in endosperm expression in their promoters (**Figure 4**). These results imply that *FvTCP* genes are likely to play an important role in strawberry fruit development and ripening. In tomato, several *SlTCP* genes, such as *SlTCP12*, *SlTCP15*, and *SlTCP18*, are preferentially expressed in tomato fruit. Moreover, these genes are regulated by RIN (RIPENING INHIBITOR), CNR (COLORLESS NON-RIPENING), and SlAP2a (APETALA2a) proteins, which are transcription factors with key roles in ripening, suggesting a role during tomato fruit development or ripening (Parapunova et al., 2014). In strawberry, *FvTCP12* and *FvTCP17*, which are homologs of *SlTCP15* and *SlTCP18*, respectively, also showed high transcript accumulation levels in fruits, indicating that *FvTCP12* and *17* might play a role in strawberry fruit development or ripening (**Figures 1A** and **5**). Additionally, ethylene is a key regulator during fleshy fruit ripening (Kumar et al., 2014). The current results demonstrate that the majority of *FvTCP* genes detected herein were downregulated or nearly unchanged following Eth treatment (**Figure 7**), suggesting that TCP transcription factors regulate strawberry fruit ripening independently of the Eth pathway.

Agro-infiltration of maturing strawberry fruit has been a useful tool for defining the gene contributions to fruit development and ripening (Hoffmann et al., 2006). However, few *TCPs* have been well characterized in terms of their potential roles in this process. In this study, agro-infiltrated fruits overexpressing *FvTCP9* revealed that the expression of a series of ripening-related genes was distinctly up-regulated (more than 3.0-fold), such as *FaQR* (quinine oxidoreductase), *FaCEL* (cellulose), and *FaEXP1/2/5* (expansin1/2/5). *FaQR*, an enzyme involved in the biosynthesis of 4-hydroxy-2,5-dimethyl-3(2H)-furanone (HDMF; Furaneol), is a key flavor compound in strawberries (Raab et al., 2006). *FaCEL* is involved in strawberry the fruit softening process via the regulation of cellulose degradation (Woolley et al., 2001). Expansins are proteins that have been demonstrated to induce cell wall extension *in vitro*, and six *FaEXP* genes have demonstrated that expansions from ripening strawberry fruit are able to catalyze extension (Harrison et al., 2001). Therefore, we speculate that *FvTCP9* might play an important role in strawberry fruit development and ripening. However, we must note that *FvTCP9* is the only gene that contains an intron in strawberry (**Figure 3**), and it was quite highly and consistently expressed during strawberry fruit development (**Figure 5**). Thus, it would be interesting to investigate whether other *FvTCP* or *FvTCP9* homologs in other plants have similar roles in fruit development and ripening.

## Conclusion

In this study, 19 *FvTCP* genes were identified in the diploid woodland strawberry *Fragaria vesca* and placed in an evolutionary context based on phylogenetic and structural feature analyses. Numerous *cis*-acting elements were found in the *FvTCP* promoter sequences, suggesting that *FvTCP* gene transcripts are controlled by a complex regulatory regime. We characterized *FvTCP* gene transcripts in different tissues and developmental stages and under various stress conditions, which suggested that *FvTCP* genes could play important roles in strawberry growth and development. In addition, we examined the subcellular localization of six FvTCP-GFP fusion proteins, which provided additional insights into their functions. Notably, transient over-expression of *FvTCP9* in strawberry fruits up-regulated several fruit ripening-related genes, indicating that *FvTCP9* might be involved in the regulation of strawberry fruit development and ripening. Taken together, genome-wide analysis of the *TCP* genes in *F. vesca* might lay the foundation for further studies unraveling the functions of strawberry *TCP* genes during strawberry growth and development.

## Author Contributions

The experiments were conceived and designed by J-YF. The experiments were performed by WW, YH, M-YC, and Y-TH. WW and J-YF analyzed the data. KG provided the ‘Heilongjiang-3’ tissue culture plantlets. WW and J-YF contributed to the writing of the manuscript. All authors read and approved the final manuscript.

## Conflict of Interest Statement

The authors declare that the research was conducted in the absence of any commercial or financial relationships that could be construed as a potential conflict of interest.

## Footnotes

1 http://planttfdb.cbi.pku.edu.cn/

2 http://blast.ncbi.nlm.nih.gov/Blast.cgi

3 http://www.ebi.ac.uk/Tools/pfa/iprscan/

4 http://pfam.xfam.org/search/

5 http://www.ncbi.nlm.nih.gov/mapview/

6 http://planttfdb.cbi.pku.edu.cn/

7 http://plantgrn.noble.org/psRNATarget/

8 http://meme-suite.org/tools/meme

9 http://gsds.cbi.pku.edu.cn/

10 http://www.ncbi.nlm.nih.gov/

11 http://bioinformatics.psb.ugent.be/webtools/plantcare/html/

12 http://www.physics.csbsju.edu/stats/

13 http://planttfdb.cbi.pku.edu.cn/

14 http://www.ebi.ac.uk/interpro/search/sequence-search

15 http://pfam.xfam.org/search/

## Supplementary Material

FIGURE S1 **Linkage group distributions of *FvTCP* genes**. Linkage group numbers are provided at the top of each linkage group. The names on the left side of each linkage group correspond to the approximate location of each *FvTCP* gene. The scale is in megabases (Mb).

FIGURE S2 **Motif sequences of FvTCP proteins identified using MEME tools**.

FIGURE S3 **Transcript accumulation pattern of 19 *TCP* genes in the diploid woodland strawberry (*F. vesca*) during different periods of subcultural propagation. (A)** Photos of strawberry subcultural propagation during the five different periods assessed (P1: original plantlet; P2: plantlet with 1–2 branch crowns, approximately 2 weeks after subculture; P3: plantlets with 3–4 branch crowns, approximately 3 weeks after subculture; P4: plantlets with 5–7 branch crowns, approximately 4 weeks after subculture; P5: plantlets with over 10 branch crowns, approximately 6 weeks after subculture). *Bar* = 1 cm. **(B)** Hierarchical clustering of the transcript accumulation profiles of 19 *FvTCP* genes during different strawberry subcultural propagation periods (original results shown in **Supplementary Figure S4**). The transcript accumulation profiles were generated by semi-quantitative PCR and were visualized as heat maps. The color scale represents relative transcript levels with increased (red) or decreased (green) transcript abundance. Genes were hierarchically clustered based on average Pearson’s distance metric and ‘average linkage’ method. *Fv18s* was used as an internal control. The experiments were repeated three times and provided consistent results.

FIGURE S4 **Transcript accumulation patterns of the 19 *FvTCP* genes during different fruit developmental stages (A)** and strawberry subcultural propagation stages **(B)** analyzed by semi-quantitative RT-PCR. *Fv18s* was used as an internal control. Lanes: **A**: S1: mature flowers with partially withered petals, S2: mature green receptacles, S3: white receptacles with green achenes, S4: half white and half red fruits, S5: fully ripened fruits. **B**: P1: original plantlet; P2: plantlet with 1–2 branch crowns, approximately 2 weeks after subculture; P3: plantlets with 3–4 branch crowns, approximately 3 weeks after subculture; P4: plantlets with 5–7 branch crowns, approximately 4 weeks after subculture; P5: plantlets with over 10 branch crowns, approximately 6 weeks after subculture.

FIGURE S5 **Transcript accumulation patterns of the 19 *FvTCP* genes under abiotic (cold, heat, drought, and NaCl) and biotic treatments (powdery mildew infection) analyzed by semi-quantitative RT-PCR**. *Fv18s* was used as an internal control.

FIGURE S6 **Transcript accumulation patterns of the 19 *FvTCP* genes exposed to hormone treatments (ABA, Eth, MeJA, and SA) and analyzed by semi-quantitative RT-PCR**. *Fv18s* served as an internal control.
