# Supplementary figures and images for: Identification and Transcript Analysis of the TCP Transcription Factors in the Diploid Woodland Strawberry Fragaria vesca
Source: Front Plant Sci. 2016 Dec 22;7:1937. doi: 10.3389/fpls.2016.01937 (PMC5177655; doi:10.3389/fpls.2016.01937)

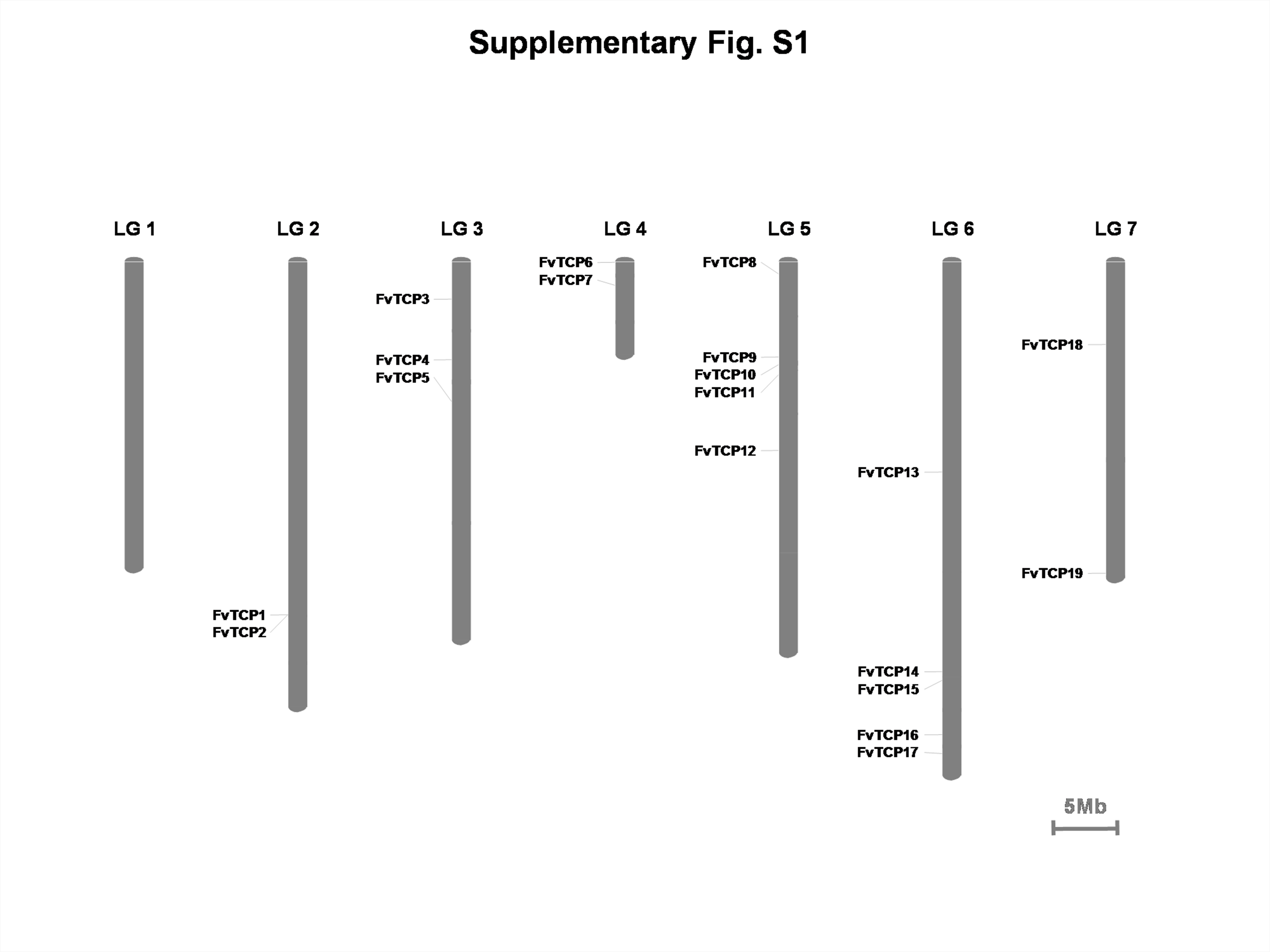

Supplement: FIGURE S1 — Linkage group distributions of FvTCP genes. Linkage group numbers are provided at the top of each linkage group. The names on the left side of each linkage group correspond to the approximate location of each FvTCP gene. The scale is in megabases (Mb). [file Image_1.TIF]

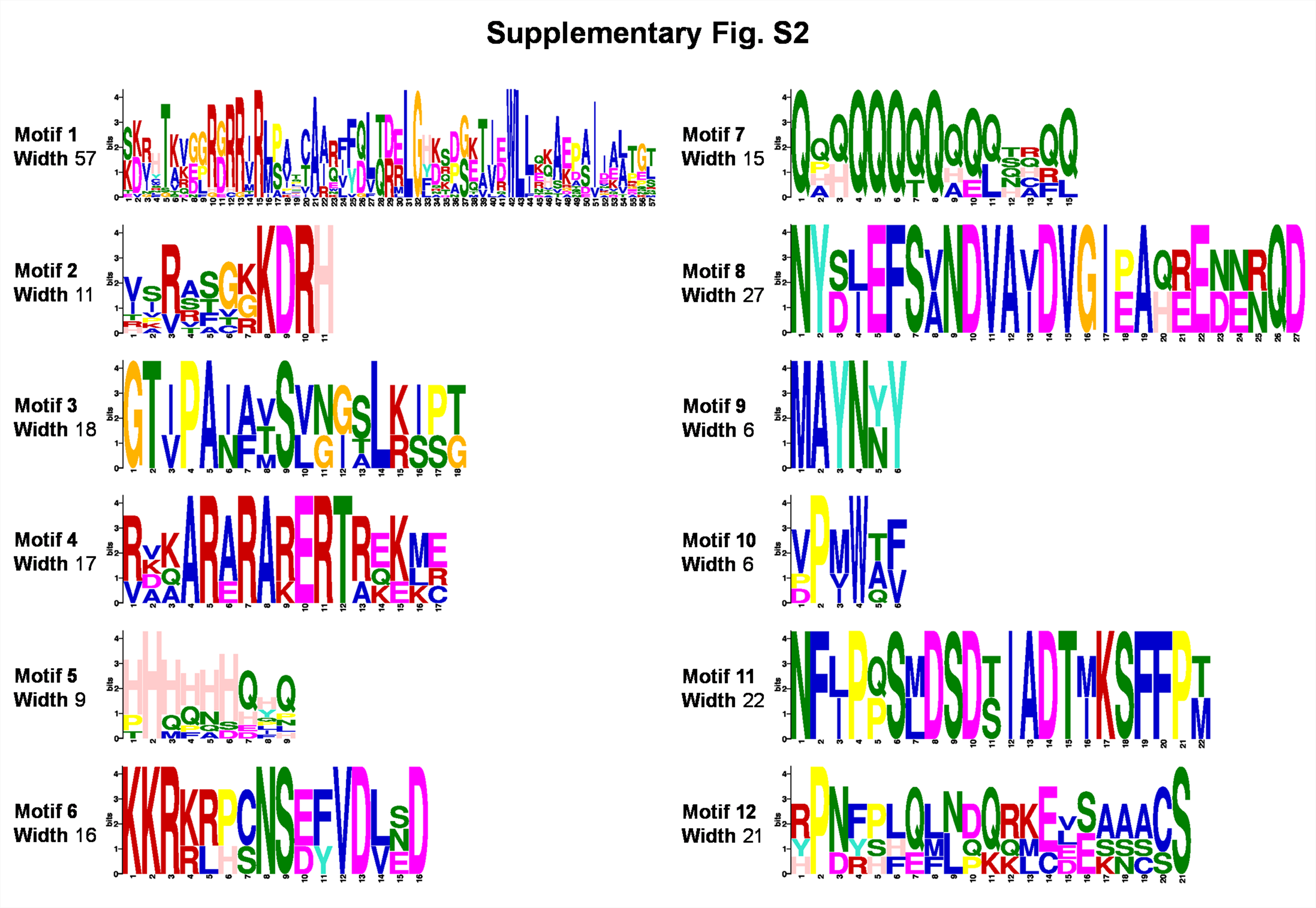

Supplement: FIGURE S2 — Motif sequences of FvTCP proteins identified using MEME tools. [file Image_2.TIF]

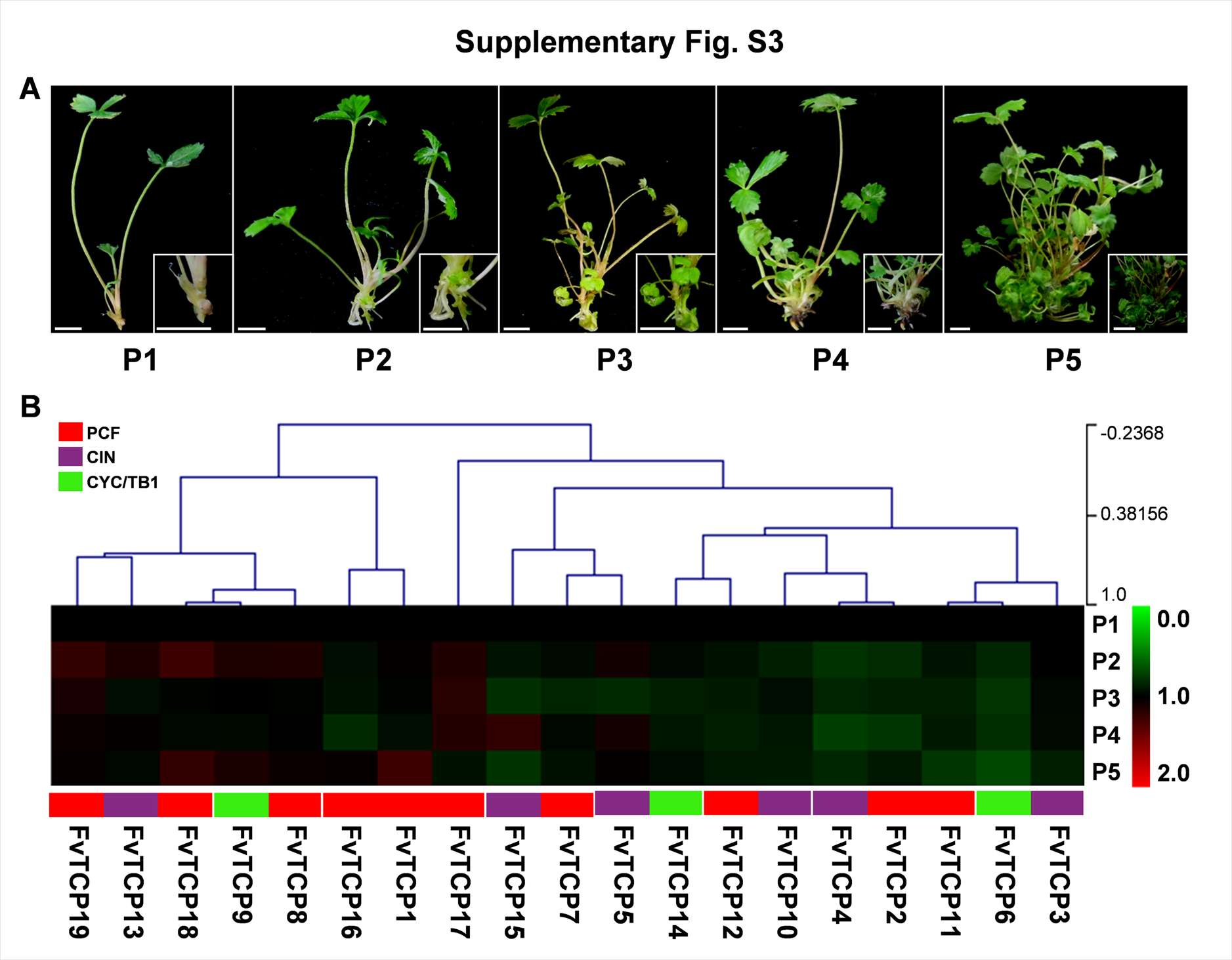

Supplement: FIGURE S3 — Transcript accumulation pattern of 19 TCP genes in the diploid woodland strawberry (F. vesca) during different periods of subcultural propagation. (A) Photos of strawberry subcultural propagation during the five different periods assessed (P1: original plantlet; P2: plantlet with 1–2 branch crowns, approximately 2 weeks after subculture; P3: plantlets with 3–4 branch crowns, approximately 3 weeks after subculture; P4: plantlets with 5–7 branch crowns, approximately 4 weeks after subculture; P5: plantlets with over 10 branch crowns, approximately 6 weeks after subculture). Bar = 1 cm. (B) Hierarchical clustering of the transcript accumulation profiles of 19 FvTCP genes during different strawberry subcultural propagation periods (original results shown in Supplementary Figure S4). The transcript accumulation profiles were generated by semi-quantitative PCR and were visualized as heat maps. The color scale represents relative transcript levels with increased (red) or decreased (green) transcript abundance. Genes were hierarchically clustered based on average Pearson’s distance metric and ‘average linkage’ method. Fv18s was used as an internal control. The experiments were repeated three times and provided consistent results. [file Image_3.TIF]

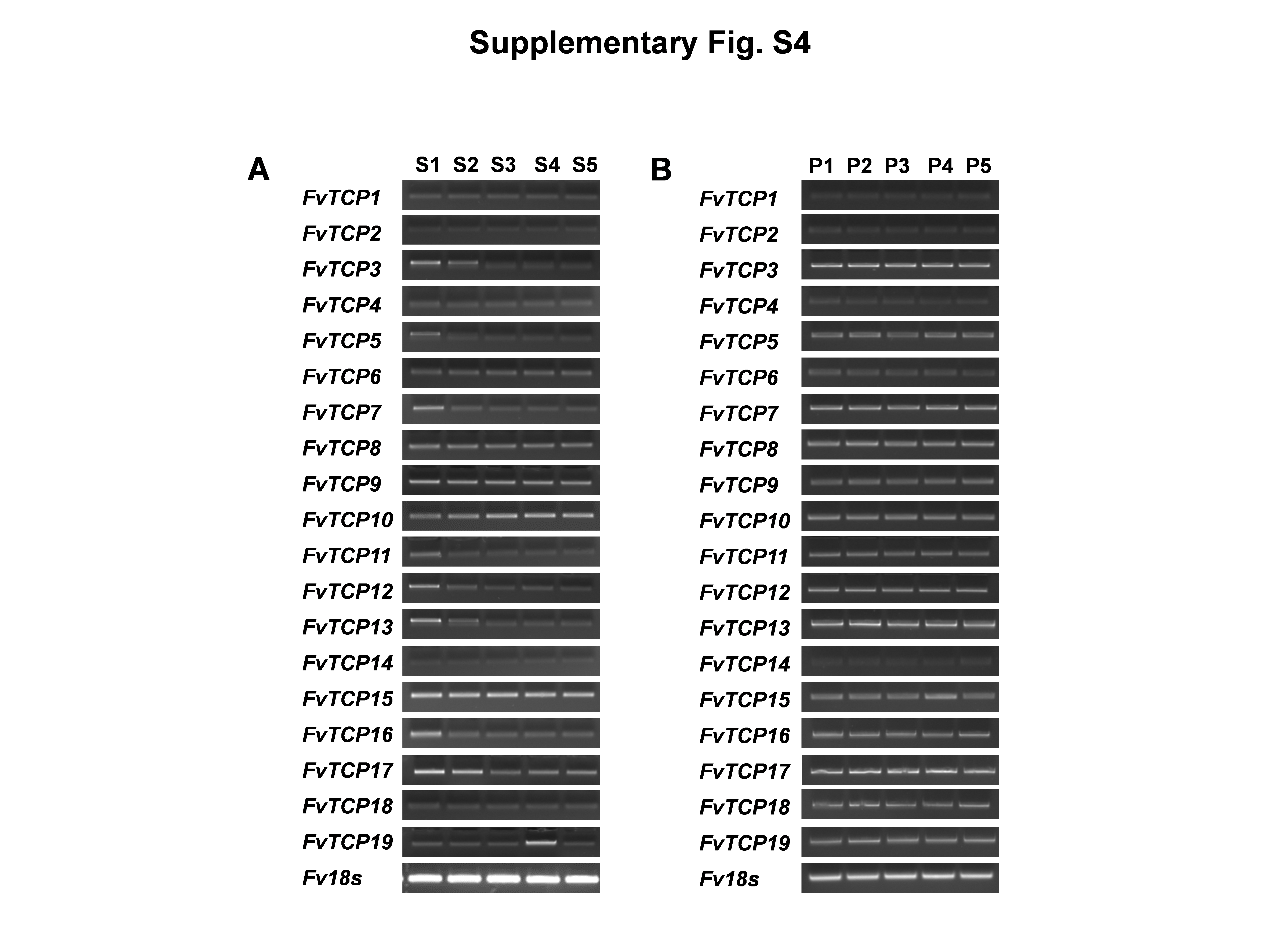

Supplement: FIGURE S4 — Transcript accumulation patterns of the 19 FvTCP genes during different fruit developmental stages (A) and strawberry subcultural propagation stages (B) analyzed by semi-quantitative RT-PCR. Fv18s was used as an internal control. Lanes: A: S1: mature flowers with partially withered petals, S2: mature green receptacles, S3: white receptacles with green achenes, S4: half white and half red fruits, S5: fully ripened fruits. B: P1: original plantlet; P2: plantlet with 1–2 branch crowns, approximately 2 weeks after subculture; P3: plantlets with 3–4 branch crowns, approximately 3 weeks after subculture; P4: plantlets with 5–7 branch crowns, approximately 4 weeks after subculture; P5: plantlets with over 10 branch crowns, approximately 6 weeks after subculture. [file Image_4.TIF]

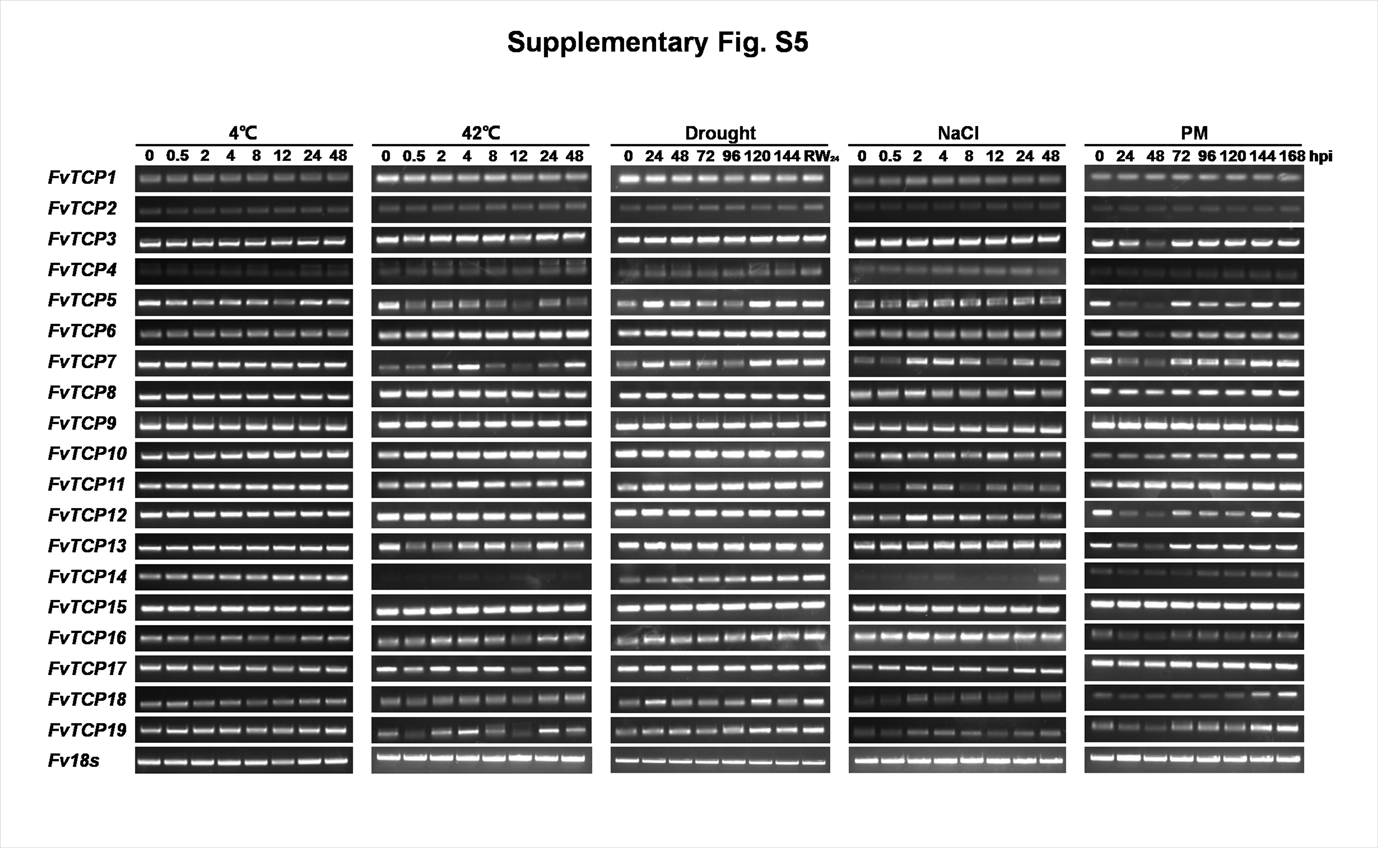

Supplement: FIGURE S5 — Transcript accumulation patterns of the 19 FvTCP genes under abiotic (cold, heat, drought, and NaCl) and biotic treatments (powdery mildew infection) analyzed by semi-quantitative RT-PCR. Fv18s was used as an internal control. [file Image_5.TIF]

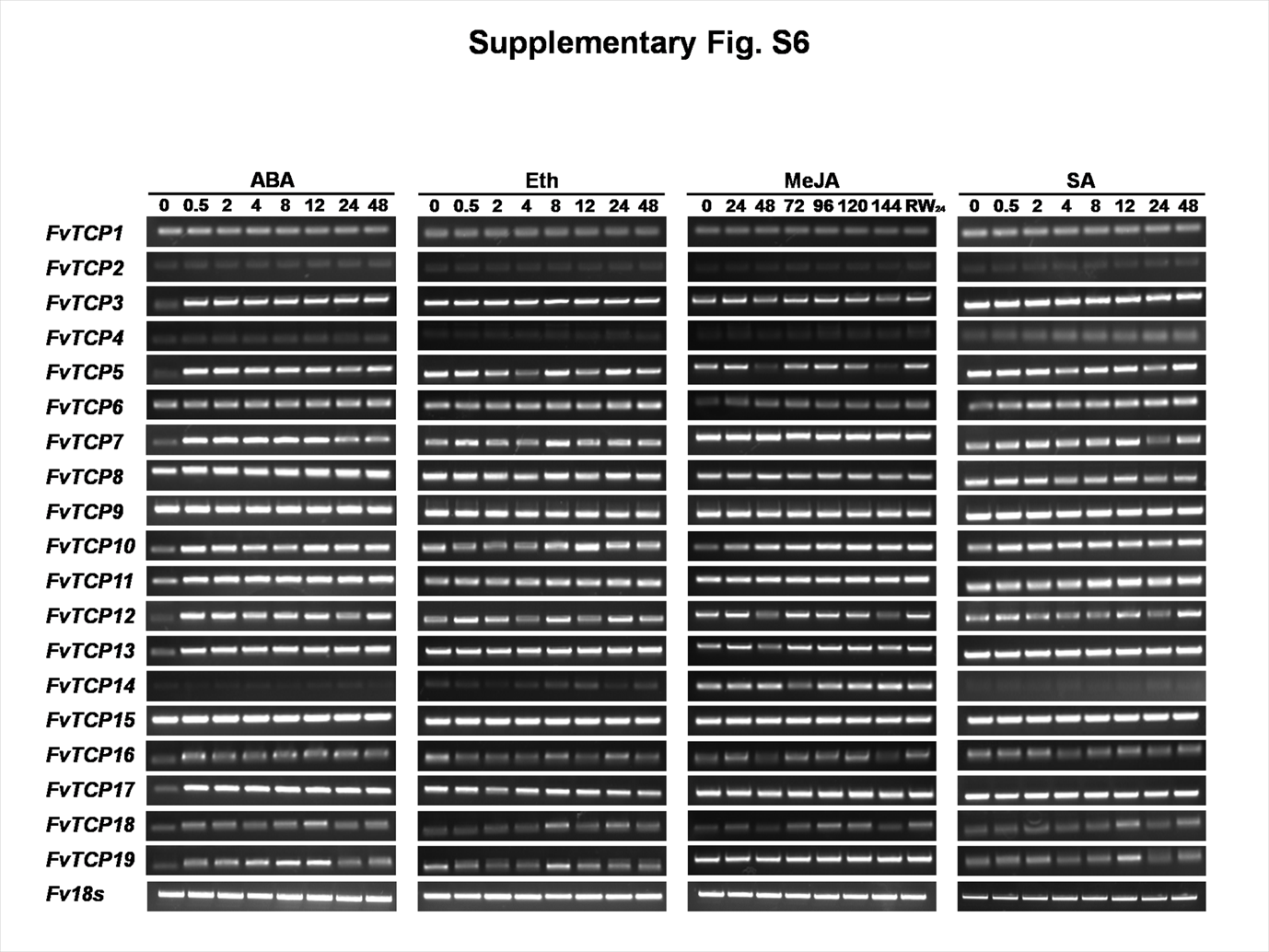

Supplement: FIGURE S6 — Transcript accumulation patterns of the 19 FvTCP genes exposed to hormone treatments (ABA, Eth, MeJA, and SA) and analyzed by semi-quantitative RT-PCR. Fv18s served as an internal control. [file Image_6.TIF]
