# Supplementary material for: Identification and Transcript Analysis of the TCP Transcription Factors in the Diploid Woodland Strawberry Fragaria vesca
Source: Front Plant Sci. 2016 Dec 22;7:1937. doi: 10.3389/fpls.2016.01937 (PMC5177655; doi:10.3389/fpls.2016.01937)
Supplement: Supplementary file 7 [file Table_1.DOC]

**Supplementary Table S1.** The primers used for the *FvTCP* genes in this study.

| **Primer Names** | **Sequence (5'-3')^1^** | **Description^2^** |
| --- | --- | --- |
| FvTCP1-qF1 | GGTGAATGCGGTATACGCTTGCC | RT-qPCR |
| FvTCP1-qR1 | GTCGCATTCTCCTCCATGTCCTCAG | RT-qPCR |
| FvTCP2-qF1 | CTCGCCTACCACCCACTTGTGCT | RT-qPCR |
| FvTCP2-qR1 | CTAACGACATTTCCTGCCTGCATATTC | RT-qPCR |
| FvTCP3-qF1 | GGCGGCAGGGAGATCAAGACT | RT-qPCR |
| FvTCP3-qR1 | GGGTTGGTCGTAACCCAACCG | RT-qPCR |
| FvTCP4-qF1 | GTTCAAGGCGGCCACATTGTGAG | RT-qPCR |
| FvTCP4-qR1 | GCCTCCTTGGTGTTGCTGAGGCT | RT-qPCR |
| FvTCP5-qF1 | CGCGGGGGCCACATTATAAGAG | RT-qPCR |
| FvTCP5-qR1 | CGCAGTCGGAGAAGCTGAAGTCTG | RT-qPCR |
| FvTCP6-qF1 | ATCAGCGTCGCGGCTTCAAAC | RT-qPCR |
| FvTCP6-qR1 | GTTGATGGCAGACCTGGACTTTGTG | RT-qPCR |
| FvTCP7-qF1 | CTGACGAGAGAGCTAGGGCACAAGTC | RT-qPCR |
| FvTCP7-qR1 | CCGCCGAACAACAAGCTGCTACT | RT-qPCR |
| FvTCP8-qF1 | CGGTGAGGAGGACCTCCACCAAG | RT-qPCR |
| FvTCP8-qR1 | GTCTTCAGCCGTGTTGTGGTGGTT | RT-qPCR |
| FvTCP9-qF1 | CCACCACCACCACCACCATGAG | RT-qPCR |
| FvTCP9-qR1 | TGTTGTTGTTGTTGCTGCTGCTGC | RT-qPCR |
| FvTCP10-qF1 | TCCAAGGATTGTTCGTGTCTCCC | RT-qPCR |
| FvTCP10-qR1 | GGTGGGAGCTTTTCAATATCATCGT | RT-qPCR |
| FvTCP11-qF1 | TCTCTAGCCATCTCCTCCGCCG | RT-qPCR |
| FvTCP11-qR1 | GTCGGACTTGTGTCCCAGCTCG | RT-qPCR |
| FvTCP12-qF1 | GCGCAGCAACCCTCCTCTAACG | RT-qPCR |
| FvTCP12-qR1 | GGAGGGAGACGGAGACGGTGG | RT-qPCR |
| FvTCP13-qF1 | CCAAAACCAGCATCAAACAACTGCT | RT-qPCR |
| FvTCP13-qR1 | GTAGCCTAGCCGGTCTTGAACATCG | RT-qPCR |
| FvTCP14-qF1 | CCAGTCTACTCGCTTTCCCTTTTCC | RT-qPCR |
| FvTCP14-qR1 | TCGCGTTTGATGAATCTCCTCCG | RT-qPCR |
| FvTCP15-qF1 | CAGCAACAGTAGCACCATGGATGAG | RT-qPCR |
| FvTCP15-qR1 | GTTGGTCGATATCATCCTTGGCC | RT-qPCR |
| FvTCP16-qF1 | CACGACTGGTGACAACTCAGGGAAG | RT-qPCR |
| FvTCP16-qR1 | AGACGGGCATGGAAGGTGTGG | RT-qPCR |
| FvTCP17-qF1 | CGTTGTGAAACCGGCGGAGATC | RT-qPCR |
| FvTCP17-qR1 | CGATTTGTGGCCCAATTCCCG | RT-qPCR |
| FvTCP18-qF1 | GAGCAAGAGCCGGAGCAGAGATC | RT-qPCR |
| FvTCP18-qR1 | GACCCGTTGACGGACATGGCTAT | RT-qPCR |
| FvTCP19-qF1 | GTGGTGAAGCAGCGGTCGTCG | RT-qPCR |
| FvTCP19-qR1 | GCCAGCGAACTCGTCGGAATCT | RT-qPCR |
| Fv18S-qF | ACCGTTGATTCGCACAATTGGTCATCG | RT-qPCR |
| Fv18S-qR | TACTGCGGGTCGGCAATCGGACG | RT-qPCR |
| FvTCP7-F1 | GAGAACACGGGGGACTCTAGAATGATCATGGAAGGTGATAATGAAAAT | cloning |
| FvTCP7-R1 | TTCTCCTTTACCCATGGTACCACGTTGGCTAGCATTTGAATGCTC | cloning |
| FvTCP8-F1 | GAGAACACGGGGGACTCTAGAATGACGTCGTATTTTGAGGATCAGG | cloning |
| FvTCP8-R1 | TTCTCCTTTACCCATGGTACCCTGGCTGCCTGGGCCGGCG | cloning |
| FvTCP9-F1 | GAGAACACGGGGGACTCTAGAATGTTTCCTTATAGCTCAAACGTT | cloning |
| FvTCP9-R1 | TTCTCCTTTACCCATGGTACCAAATCCATTATGAGTACTACTGTTGT | cloning |
| FvTCP13-F1 | GAGAACACGGGGGACTCTAGAGTGAGACCTATCCACCAAATTAAAGAC | cloning |
| FvTCP13-R1 | TTCTCCTTTACCCATGGTACCGTGGTGGTTGGAGTTGGGAGAGG | cloning |
| FvTCP15-F1 | GAGAACACGGGGGACTCTAGAATGATTAGGAGTCCCAATACTAATG | cloning |
| FvTCP15-R1 | TTCTCCTTTACCCATGGTACCCCTAGAAGGAGGCTTCTGGTCTTTA | cloning |
| FvTCP17-F1 | GAGAACACGGGGGACTCTAGAATGGATCCCAAGGCCTCAAAAC | cloning |
| FvTCP17-R1 | TTCTCCTTTACCCATGGTACCCTGTCCTGAGCCTTGAGAATCATC | cloning |

1 Restriction sites are indicated in red.

2 The type of experiment for which the primers were used is indicated in brackets (RT-qPCR: reverse transcription quantitative PCR).
