# Supplementary material for: Identification and Transcript Analysis of the TCP Transcription Factors in the Diploid Woodland Strawberry Fragaria vesca
Source: Front Plant Sci. 2016 Dec 22;7:1937. doi: 10.3389/fpls.2016.01937 (PMC5177655; doi:10.3389/fpls.2016.01937)
Supplement: Supplementary file 9 [file Table_3.DOC]

**Supplementary Table S3.** The primers used to analyze the expression of ripening-related marker genes in the transient over-expression of *FvTCP9* in strawberry fruits.

| **Gene** | **Forward primers (5’-3’)** | **Reverse primers (5’-3’)** | | **Genbank accession numbers** | |
| --- | --- | --- | --- | --- | --- |
| *FaC4H* | ACGCTCAACAGAAAGGAGAGAT | | TTCGGGGTGGTTCACAA | | DQ898278.1 |
| *Fa4CL1* | CGTAGACCCTGAAACTGGTG | | GTGTAGCCATCCTTCCTTGTC | | gene15877 |
| *Fa4CL2* | ACGAATCCCTTCCCAGAAA | | TCTTTGTCTATGGTCCTCTCAGTC | | gene05255 |
| *FaCHS* | CATACCCCGACTACTACTTTCGT | | CGCACATACTGGGATTCTCTT | | AY997297.1 |
| *FaCHI* | AGCGAAAGCCATTGAAAAGT | | CATTTGGTGATTGTGTGAAGAG | | AB437286.1 |
| *FaF3H* | CTTTCGTGGTGAATCTTGGAG | | TCGCTATGGACAACCTGCT | | AB665441.1 |
| *FaDFR* | ACCCTGAGAACGAAGTGATAAAG | | TAAACACCACCCTCCGAACT | | AY695813.1 |
| *FaUFGT* | TAGAGGATGTGTGGAAGATTGGT | | CTGTTGTGCGAGTTGTTTTAGTG | | AY575056.1 |
| *FaANS* | CTTGGCTTGGGATTAGAAGAAG | | TGAGGGCATTTTGGGTAGTAGT | | AY695818.1 |
| *FaPE* | GGTTTCTACTGGTGCTGGTTTT | | CTCGGACTGTATCGTGTTGC | | AY324809.1 |
| *FaPL* | TCAACTCGTCAATGGCAGAC | | GAATGCTCGTATCAACCAGAGA | | U63550.1 |
| *FaPG* | GCAAGTAGAGTCGCACAGTTTT | | TCAGTATTAGGCTTCCCACCA | | DQ45899.1 |
| *FaCEL* | GCTCTGTTTTGCCTGGACTT | | GCGTGGCTTAGATAGTTGGAAT | | AF051346.1 |
| *FaGAL1* | CAAAAGGGTGGAGCATTCAT | | CCAGAGGAGCATCGTAATCATA | | AJ278703.1 |
| *FaGAL2* | GAGGGAAGGAACGATTTGG | | ACAGAGGAAAGCCACCGTAA | | AJ278704.1 |
| *FaXYL1* | ATGGAAAGCCTACTTGTGCTG | | CTGGTGTAATGTTGTTGGTCGT | | AY486104.2 |
| *FaEXP1* | AGGACGGAGTTGGATTGC | | TGAGCGTGAGCGTGAAG | | AF163812 |
| *FaEXP2* | GTATCGTCCCCGTCTCATTC | | AGTAGGAGTGCCCGTTGATT | | AF159563 |
| *FaEXP3* | TCACTGCCACTAACTTCTGC | | TTATGCCTCCTGCTCTCCT | | AF226700 |
| *FaEXP4* | TACAGCCAGGGGTATGGAAC | | GTTAGGGAGGGCATTGTTTG | | AF226701 |
| *FaEXP5* | CTTCTTACTCTGACCCCATTGTTT | | TGTGCTTAGTGCTGCTGTGTT | | AF226702 |
| *FaQR* | CACTGACTCTCCCCTACCTACAAT | | ATACACTTCATCCCCCACCTTA | | AY048861.1 |
| *FaOMT* | CACCAAGGGAGTTGTCCAT | | GTTGAAAGCATCACAGCAGAC | | AF220491.1 |
